# Becoming Sustainable, The New Frontier in Soft Robotics

**DOI:** 10.1002/adma.202004413

**Published:** 2020-12-18

**Authors:** Florian Hartmann, Melanie Baumgartner, Martin Kaltenbrunner

**Affiliations:** ^1^ Soft Matter Physics Institute of Experimental Physics Johannes Kepler University Linz Altenberger Strasse 69 Linz 4040 Austria; ^2^ Soft Materials Lab Linz Institute of Technology LIT Johannes Kepler University Altenberger Strasse 69 Linz 4040 Austria; ^3^ Institute of Polymer Science Johannes Kepler University Altenberger Strasse 69 Linz 4040 Austria

**Keywords:** biodegradable, electronic skins, green electronics, green materials, soft robotics, sustainability

## Abstract

The advancement of technology has a profound and far‐reaching impact on the society, now penetrating all areas of life. From cradle to grave, one is supported by and depends on a wide range of electronic and robotic appliances, with an ever more intimate integration of the digital and biological spheres. These advances, however, often come at the price of negatively impacting our ecosystem, with growing demands on energy, contributions to greenhouse gas emissions and environmental pollution—from production to improper disposal. Mitigating these adverse effects is among the grand challenges of the society and at the forefront of materials research. The currently emerging forms of soft, biologically inspired electronics and robotics have the unique potential of becoming not only like their natural antitypes in performance and capabilities, but also in terms of their ecological footprint. This review outlines the rise of sustainable materials in soft and bioinspired robotics, targeting all robotic components from actuators to energy storage and electronics. The state‐of‐the‐art in biobased robotics spans flourishing fields and applications ranging from microbots operating in vivo to biohybrid machines and fully biodegradable yet resilient actuators. These first steps initiate the evolution of robotics and guide them into a sustainable future.

## Graphical abstract

In future, robots will be seamlessly integrated in everyday life and—as much as consumer electronics is omnipresent today—assist in a myriad of tasks. However, environmental concerns at their end‐of‐life phase necessitate sustainable solutions for actuators, energy storage, electronics, and all involved materials. Soft and bioinspired robotics offer a remarkably unique potential to succeed this challenge.

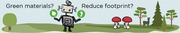

## 1 Introduction

A world affected by climate change and prevalent waste production demands environmental impact as a key metric of technological innovations. Tech disposables in particular represent a rapidly growing fraction of our garbage, accumulating in over 100 000 tons per day.^[^ ^1^ ^]^ End‐of‐lifetime appliances such as consumer electronics are typically trashed, as the various product designs and material compositions are difficult to recycle yet are cheaply produced. Additionally, the unsustainable use of rare and often toxic materials poses an environmental threat when released into nature due to improper treatment or landfilling.^[^ ^2^ ^]^ Easy to recycle device designs, low‐cost and renewable materials, and biodegradable or transient systems are promising approaches toward technologies with a closed life cycle and establish new opportunities across different fields from medicine and environmental monitoring to security and intelligence applications.^[^ ^3^ ^]^

Current developments in robotics that focus on safe human–machine interaction, swarm robotics, and untethered autonomous operation are often inspired by nature's diversity.^[^ ^4^ ^]^ The complexity we find in nature drives scientists from various fields to establish soft and lightweight forms of robots that aim to replicate or mimic the fluent motion of animals or their efficient energy management.^[^ ^5^, ^6^ ^]^ In future, the increased integration of such soft robots in our everyday life raises, in close analogy to consumer electronics, environmental concerns at the end of their life cycle. Again, we can learn here from nature and design our creations sustainably and mitigate the problems of currently used technology. In contrast to standardized industrial robots that are already integrated in recycling loops, bioinspired robotics will find various applications in diverse ecological niches.^[^ ^7^ ^]^

Possible examples range from soft healthcare machines that support elderly people in their everyday lives to robots that first harvest produce and afterwards become compost for next season's plants. Current demonstrations with transient behavior include elastic pneumatic actuators,^[^ ^8^ ^]^ wound patching millibots operating in vivo,^[^ ^9^ ^]^ robot swarms for drug delivery,^[^ ^10^ ^]^ or small grippers that are controlled by engineered muscle tissues.^[^ ^11^ ^]^ These developments benefit from major research activities toward bioresorbable electronic devices,^[^ ^12^ ^]^ which are mainly explored for the biomedical sector, and sustainable energy storage technology,^[^ ^13^ ^]^ seeking to resolve environmental concerns for the increasing demand of energy for mobile appliances. Bringing those fields together will be the future challenge for autonomous robots, whether their development focuses on performance, sustainability, or both. The efficient integration of actuators, sensors, computation, and energy into a single robot will require new concepts and ecofriendly solutions, and can only be successful if material scientists, chemists, engineers, biologists, computer scientists, and roboticists alike join forces.

In this review, we highlight materials, fabrication approaches, and design routes for environmentally friendly bioinspired robots and their components. We focus on sustainable device concepts, nontoxic, and low‐cost fabrication processes, and benign materials that are biodegradable or from renewable resources, to address the challenging needs of our time. The review starts with a discussion of sustainability and summarizes various approaches that enable technology with reduced environmental impact. Focusing on soft and lightweight forms of robotics, we then compare biodegradable polymers—from elastomers to bioplastics—and regrowing resources for the main robotic body. For each component of typical autonomous robots, we review environmentally friendly sensors, computation, and control tools and present promising candidates for energy harvesters and storage systems, from solar‐ and biofuel cells to batteries. Finally, we present a selection of current soft robotic demonstrations that use frugal material approaches and degrade with a positive impact on the environment.

## 2 Sustainable Approaches for Soft Robotics

**Figure 1:**
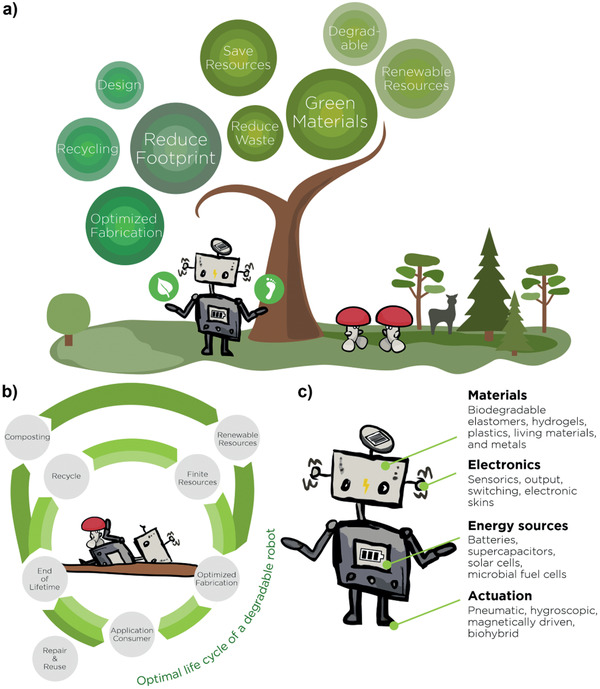
Sustainable robotics development. a) Strategies toward ecofriendly robotics include several pathways from material approaches to optimized design and fabrication. b) The optimal life cycle of a robot is a closed loop, which c) targets all robotic components from actuation to energy supply.

Sustainable materials development for robotics mainly targets two scientific questions. First, can we employ new materials and resources that contribute to a more sustainable future? and second, how can we use or modify existing materials to reduce their ecological footprint on the environment? Solutions to the first question include high performance materials with increased durability, materials from renewable sources, or biodegradable ones, with the target to save valuable resources or to reduce waste. The same goals apply for solutions to the second question, but instead of developing new materials they target fabrication processes, recycling, and product designs (**Figure** **1**). Sustainability in robotics covers many facets, approaches, and solutions from which we discuss renewable resources, recycling and biodegradability within this section.

### 2.1 Renewable Resources

In contrast to finite resources such as fossil fuels, nuclear fuels, and rare earth metals, renewable materials are either constantly available or are naturally replaced within reasonable timescales. In an ideal sustainable usage, the material/energy consumption rates equal the regeneration rate of the resources. Autonomous robotics potentially benefit from renewable resources more than other technologies, by harvesting energy from solar power or tidal waves and by replacing broken body parts with spare parts that regrow in nature.

Solar power, a long‐time standard for space exploration robots, provides unlimited energy supply that can be stored in a robot's battery to deliver constant power over an extended period of time. The smaller and lighter a robot is, the more efficient it is to use solar power over fuel energy, as robots only need to carry collectors but not the fuel itself. Extremely lightweight solar panels for example are able to deliver high amounts of power (23 W g^−1^) without adding much weight to the robot.^[^ ^14^ ^]^ Instead of fossil‐based plastics the robotic body can be built from plant‐based materials. Green composite materials are promising candidates for hard but lightweight parts, not only for robots but mobile machines in general.^[^ ^15^ ^]^ For electric cars, lightweight natural fiber composites with eligible mechanical properties could replace dense synthetic materials for interior and exterior components and balance the increasing weight of batteries. For the rising machine–human interaction, elastomers synthesized from biomaterials can render soft grippers or (robotic) soft electronic skins (e‐skins) analogous to biological designs. For many electronic components, carbonized biomass can be used as electron conductive substitute replacing metals or participate in the electrochemical reactions of batteries and supercapacitors.^[^ ^16^, ^17^ ^]^

**Figure 2:**
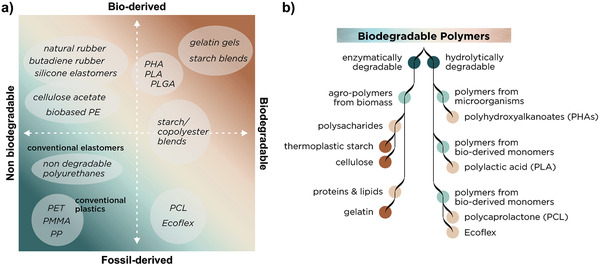
Classification of biodegradable polymers. a) Elastomers and plastics divide into four sectors between fossil‐ or bioderived and non‐ or biodegradable. b) Each polymer is classified depending on its degradation mechanism and source.

However, the utilization of renewable materials mainly targets resource issues but not necessarily waste issues. Vulcanized natural rubber, although naturally derived, does not degrade in reasonable time and requires waste treatment and recycling (**Figure** **2**a). Renewability and biodegradability, or recycling must be optimized as a whole to yield a sustainable technology with a positive impact on resources and waste.

### 2.2 Recycling

For technology that has to meet high performance standards—complementary metal‐oxide‐semiconductor (CMOS) chips or Bluetooth communication—renewable or biodegradable solutions are still elusive. Here, recycling represents a feasible approach toward a more sustainable use of technology. Recycling however should be seen as the transformation of waste to a valuable (similar) product. The generation of energy through combustion of waste is termed recycling as well, although it is merely sustainable as it consumes resources and increases CO_2_ emission.

In general, material‐, device‐, or robot recycling follows economical viewpoints: A product is more likely to be recycled if recycling is cheaper than the fabrication costs of a new one. Therefore, the ideal recycling process must be cost‐effective, technologically easily achievable, integrated in closed production‐recycling loops, target valuable materials and require little energy. Lead‐acid batteries (car batteries) are an example for efficient recycling. As such batteries have a standardized simple architecture, they can easily be dismantled and recycled.^[^ ^18^ ^]^ Trained technicians that replace the batteries close the life‐cycle loop by sending broken batteries back to the manufacturers.

The recycling of other electrical waste (e‐waste) is often problematic and less easily achievable as the architecture and material composition of integrated circuits, Li‐batteries, or displays widely deviate. To reduce recycling costs, e‐waste is often shipped to developing countries such as Ghana, where improper e‐waste processing puts workers and residents at risk.^[^ ^2^ ^]^

To render robotics sustainable, recycling must be already included in the design phase. A successful recycling scheme requires the individual robotic materials to be easily separable to enable uncomplicated reuse, exchange, and upgrade of robots. While more readily achievable for classical robots, which are often assemblies of standardized electronic parts and actuators, this can be challenging for soft robots that feature various actuation principles and materials. Yet, soft robots benefit from less complex material arrangements. Pneumatically driven soft robots, for example, have combined actuators and bodies. Therefore, the complexity of recycling a whole robotic body with many actuators (that consist of various components themselves) is reduced to the recycling of a single material. Likewise, the less stringent requirements of control feedback allow e‐skins with reduced materials complexity. A fruitful approach is to incorporate self‐healing materials or concepts for soft robots that restore materials functionality autonomously. Tan and co‐workers developed a stretchable optoelectronic material for stretchable electronics and soft robotics with light emission and feedback sensing, which autonomously self‐heals after puncture.^[^ ^19^ ^]^

Another sustainability approach is to use less material by design.^[^ ^20^ ^]^ Autonomous robots benefit twofold from lightweight materials/component designs, aiming to first reduce the weight and increase operation time, and second minimize environmental impact by reducing the total amount of waste. Zero waste robotics will be finally possible by fully biodegradable materials.

### 2.3 Biodegradation

Biodegradable materials are a promising material class for sustainable technology. In the ideal case, a material breaks down into smaller, environmentally benign components, which are metabolized by bacteria or enzymes at timescales that are comparable to typical waste processing. Moreover, the degradation process should start at the end‐of‐life phase of a device, initiated by a trigger, and happen at a controlled rate and under feasible environmental conditions. Across literature, biodegradability is not clearly defined and handled, in particular when it comes to multicomponent/material devices. For biodegradable electronics, for example, it is often the case that not all components are biodegradable or they degrade at distinctly different rates. Bao and co‐workers differentiate materials with transient behavior (type I) that disintegrate into sufficiently (macroscopically) small components and biodegradable materials (type II) that undergo complete chemical degradation into microscopically small molecules.^[^ ^21^ ^]^

Transient electronics, assembled from type I materials, play a major role in the biomedical sector. Implantable or edible devices are designed to reside in our body, as they monitor cardiac pressure, glucose levels, or neural activities. The degradation of these devices must be accomplishable under physiological conditions to render truly bioresorbable devices. Therefore, the lifetime of all materials should be limited to timescales comparable to healing of human tissue or regeneration processes, and each degradation product must be noncytotoxic. Such material designs are likewise a promising route for microbots that operate in vivo, for wound treatment or drug delivery applications. Outside the body, biodegradable materials enable secure systems that vanish after their operation, to avoid plagiarism, espionage, or abduction of critical technology.

Biodegradable robotics and electronics (type II) require a complete metabolization of all constituents. Here, it is not sufficient that materials break into smaller units but they must be converted into biomass or gasses by microorganisms. In addition, materials that degrade into bioderived small molecules offer intrinsic biocompatibility and recyclability, while delivering energy back to nature. Such technology finally may resolve critical problems with e‐waste and simultaneously offers a transformation of conventional robotics toward creative solutions that comprise the whole life cycle of technology.

As the areas of application, environments of operation, and timescales of degradation can largely vary for type I or II technology, it is important to correctly report all three specifications for the materials we use. Implanted devices must degrade under physiological conditions resembling the target environment of our body; produce harvesting robots should decompose in organic waste and compost; maritime fish robots require materials that disintegrate in seawater. Immersing a material into an unsuitable environment may not cause any degradation at all, even though it is specified biodegradable. This misconception is unfortunately rather common among reports of biodegradable materials and notably illustrated by Bagheri and co‐workers.^[^ ^22^ ^]^ They immersed typical biodegradable polymers such as polylactic acid (PLA), polycaprolactone (PCL), and poly(3‐hydroxybutyrate) (P3HB) in seawater to study their degradation. Surprisingly, they found that those polymers barely degrade over timescales of 400 d with mass loss of less than 10%. This is also the case for the elastomer Ecoflex used by the soft robotics community. This polymer is 100% fossil‐based but fully decomposes in ≈80 d under industrial composting conditions.^[^ ^23^ ^]^ Cellulose, for example, requires about 50 d under the same conditions. In seawater, temperature, microorganisms, and availability of oxygen largely differ from those found in compost, extending the degradation time of Ecoflex by multiple orders of magnitude.

While there are standards also for biodegradation in sea water, the most common standards that certify (packaging) polymers as biodegradable target degradation in industrial composting facilities. The worldwide applicable standard is the ISO 17088 norm. It is based on the European EN13432 and American ASTM 6400‐04 standards and is effective since 2008. In essence, biodegradation tests monitor the CO_2_ evolution of polymer/compost mixtures at optimum humidity and oxygen conditions at 58 °C, and the standards specify the pass levels.

Where industrial composting is no option, biodegradable materials are needed that also disintegrate in less controlled environments. Tech‐waste that is disposed of via household composts or somewhere in nature needs to vanish under milder conditions, yet at equally fast rates. Additional declarations for biodegradable materials in electronics or robotics should indicate that the robot, after fulfilling its purpose and reaching the end of its life cycle, can simply be thrown away without taking care of environmental conditions or be left at the place of his “death.” Therefore, it requires research, standards, and specifications to progress toward materials that allow individual‐based waste management.

## 3 Materials

The architecture of typical hard robots can be reduced to a mechanical system consisting of actuators and joints, powering units, and control electronics. All these components are well separated inside the robot's body and serve a specified purpose or function. In nature, locomotion, energy storage, sensing, and computation are closely intertwined and built around the same soft material design. These blurred lines also exist for soft robotics, with the result that much of a soft robot's function depends on material design rather than on the combination of electromechanical systems.^[^ ^24^ ^]^ Likewise, environmental concerns (and even economical viewpoints such as processability and price) are at first a material science question. This section addresses the material requirements for soft robotics, gives an overview on (biodegradable) material approaches, and reviews strategies to improve the material performances.

### 3.1 Selecting Suitable Materials

**Figure 3:**
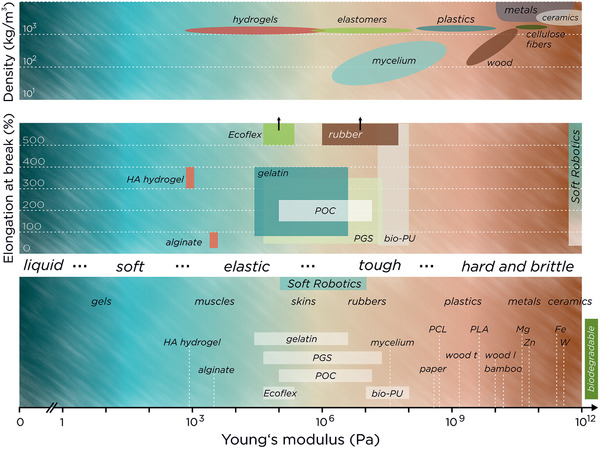
Mechanical properties of biomaterials and reference materials.

Designing the architecture of soft robots depends on the available materials and their properties. Typically, researchers employ silicone elastomers as they are soft and highly stretchable, plastics (such as polyethylene terephthalate (PET), biaxially oriented polypropylene (BOPP), or polyimide (PI)) as lightweight support for electronics and actuators or as reinforcement of body parts similar to a skeleton, and metals or inorganic semiconductors as electronic components. These different material classes span a diverse spectrum of material properties and can be conveniently differentiated based on their mechanics from soft to hard. Focusing on sustainable robotics, we first have to think which sustainable materials are available in this spectrum, second, can we replace classic material choices without sacrificing performance, and third what functions do sustainable materials provide beyond classic ones. Reviewing the literature, a broad selection of bio‐derived and synthetic materials is available—from biodegradable soft hydrogels, bioplastics and wood, to transient metals and semiconductors—to build new sustainable architectures for soft robotics (**Figure** **3**). However, before these materials can be employed in our soft robots we need to define material requirements that address both sustainability and performance.

#### 3.1.1 Mechanics

The function of materials for soft or hard (lightweight) robots and their hybrids mainly depend on their mechanical properties. These can span orders of magnitude, such as the Young's modulus (*E*) of human building blocks ranges from 1 to 10 kPa for brain tissue,^[^ ^25^ ^]^ skin (*E*: 0.1–1 MPa),^[^ ^26^ ^]^ tendon (*E*: 0.5–1 GPa),^[^ ^27^ ^]^ to bone with a Young's modulus in the range of 10 GPa.^[^ ^28^ ^]^ For soft robotic actuators, elastomers require a Young's modulus in the range from 0.1 to 10 MPa and a stretchability beyond 200% ultimate strain, as such strains typically occur for pneumatic actuators or dielectric elastomer actuators. The higher the (repeatable) stretchability is, the less probable is the occurrence of material failure. The accuracy of reversible actuation mainly depends on the shape recovery of the elastomer under cyclic stretching and material fatigue. Structural support materials such as plastics exhibit a Young's modulus in the range of 1–5 GPa with a stretchability of a few percent strain, similar to human tendons. Thermosets and thermoplastic materials are favorably used for robotic parts due to versatile fabrication methods and processability.

#### 3.1.2 Fabrication

While the gold standard for industrial processing of elastomers and plastics is injection‐molding or extrusion, cast‐molding or 3D‐printing are commonly employed fabrication techniques in soft robotics labs. Therefore, the processability of materials can be divided into three criteria: 1) Materials are castable into sheets or simple 3D structures (cubes, balls, tubes). 2) Fabrication of complex 3D structures like enclosed tubes, balloons, or functional architectures is possible. 3) Material is form‐stable meaning that shrinkage (or expansion) is below 10 % of volume to enable reliable manufacturing. Materials that fulfill all three criteria are considered excellently processible, materials that fulfill two of them are medium/moderate processible, others have low processability.

#### 3.1.3 Material Costs

Feasible material costs are the key to enable widespread application and industrial fabrication of sustainable technology, in addition to performance and processability. The total costs include the material costs of all constituents, fabrication, and production time, and equipment/labor costs. Biomaterials are usually inexpensive raw materials, yet—with the exception of food grade materials—less distributed than mineral oil. Research toward sustainable soft robotics must include material costs as essential parameter to render such technologies competitive. Extended fabrication times such as material synthesis or incompatibility with existing fabrication lines additionally increase costs and may hamper widespread market introduction.

#### 3.1.4 Biodegradability and Shelf Life

Biodegradable materials greatly reduce the environmental impact at the end of a robot's life cycle. As discussed in Section 2.3, the degradation time has to fit the desired application, the environment of waste disposal, and the prevailing conditions. In general, however, materials require short degradation times to not impose a threat to wildlife when carelessly disposed. Degradation times of a few months in sea water would already greatly reduce waste disposed in oceans. We categorize degradation times below 10 d as readily biodegradable, degradation times of several weeks as moderately sufficient and materials that need years to degrade as barely biodegradable. In contrast, materials should not immediately start to degrade after their fabrication and therefore require a long shelf life. Shape as well as mechanical, electrical, and optical properties needs to be maintained during the robot's operation, but their degradation should start when they are disposed. This dilemma is resolved by built‐in degradation triggers such as enzymes, heat, or light, which can naturally occur in different environments or are artificially induced.^[^ ^29^ ^]^ Designing robots that, for example, operate in ambient conditions but degrade in organic waste or seawater, can be a frugal route toward triggered degradation that only requires a change of environment. If operation and disposal environment are the same, the utilization of coatings that tune the robot's lifetime might be a fruitful approach. Among biodegradable materials, shelf life is a rather unexplored material parameter, yet a necessary requirement for robotic materials. Elastomers require a shelf life of at least one month to enable—the sometimes timely—assembly of soft robots and their operation. Even a shelf life of half a year is only sufficient for intermediate performance and needs to be extended to storage times of over a year which is a promising timeframe to guarantee viability. We here define selection criteria for shelf life: <1 month, not sufficient; <6 months, moderately sufficient; >6 months, sufficient.

### 3.2 Biodegradable Elastomers and Hydrogels

In analogy to soft biological systems, soft and continuum robots benefit from their compliant and adaptive bodies when interacting with their environment. They achieve an impressive range of functionality by utilizing deformable elastomers to mitigate the implementation of complex control loops, sensors, or machine learning based training. Additionally, elastomers can be used as single material for actuators, sensors, and body in all‐in‐one solutions and open paths to sustainable design approaches through recycling, renewable materials, and biodegradability. However, uniting sustainable aspects with the demands for highly stretchable, durable, low‐cost, and easy to process robotic materials is challenging. In this section, we highlight some material approaches that cover elastomeric materials from synthetic polymers to bioderived hydrogels.

Ecoflex, for example, is a biodegradable aliphatic–aromatic copolyester, which is often used in soft robotics and synthesized from 1,4‐butanediol, adipic acid, and terephthalic acid.^[^ ^23^ ^]^ Although this polymer is completely fossil‐based, it is entirely biodegradable when industrially composted as there are no toxic byproducts or residues, which could accumulate in organisms. Yet, as discussed in Section 2.3, the degradability of Ecoflex is limited to industrial composts, as it proves to be very resilient when disposed in household‐trash, natural environments, or seawater. For soft robotics, Ecoflex provides high stretchability ranging from 600% to 900% strain at a low Young's modulus ranging from 50 to 100 kPa, while being reversibly stretchable with low hysteresis.^[^ ^30^ ^]^ In the fabrication process of robotic parts, Ecoflex is typically molded after mixing a two‐component system and cured at room temperature (faster curing at higher temperatures). Combining (semi‐) cured objects is achieved by using primer chemistry or plasma bonding to form closed tubes, complex geometries, or modulus graded composites.^[^ ^31^ ^]^

Higher levels of sustainability are realized by using bioderived monomers instead of fossil‐based ones. Biodegradable polyurethanes (bio‐PUs) provide a tunable material chemistry platform for high‐performance materials. Bio‐PUs are intensively researched for biomedical applications, such as long‐term implants, due to their good biocompatibility, high tunability, and good processability. Synthesized as either thermoplastics or thermosets, polyurethanes are easy to process by injection molding, extrusion, or 3D‐printing. With the progresses in the field of tissue engineering, bio‐PUs were developed as implants for tissues with slow healing rates and are reviewed in detail by Chen et al.^[^ ^32^ ^]^ Such bio‐PUs likewise have slow degradation rates and are usually resorbed in physiological conditions within several weeks to months. Biodegradability as well as mechanical properties are tuned during synthesis by altering the ratios or types of soft segments, hard segments, and chain extender. Polyols such as PCL or PLA are typically used as soft segments and copolymerized with isocyanates as hard segments.^[^ ^33^ ^]^ The structure or ratio of soft and hard segments defines the mechanical properties of bio‐PUs with typical Young's moduli above 10 MPa. The chain extender enhances the degradation of the hard segments by making them susceptible for enzymes. Skarja and Woodhouse utilized a phenylalanine‐based chain extender to mediate enzyme‐driven (chymotrypsin) biodegradation of bio‐PUs with degradation rates of ≈10 wt% per month.^[^ ^34^ ^]^ Common for bio‐PUs are extremely high ultimate strain values of several hundred percent to above 1000%. However, these extensions stem from irreversible deformations of the elastomers. Typically, such bio‐PUs are reversibly stretchable to 50% strain and below, excluding them as candidates for soft robotics. Recent approaches address this issue by introducing shape memory effects into the polymer and achieve 98% shape recovery after 15 s at body temperature.^[^ ^35^ ^]^ Yet, further progress is required to render more reversible stretchable bio‐PUs, with lower Young's modulus and faster degradation rates.

Chemically crosslinked yet biodegradable polyesters from bioderived reagents such as poly(polyol sebacate) (PPS) represent a promising material solution for soft robotics. As most biodegradable elastomers, PPSs were originally developed for tissue engineering applications, due to excellent biocompatibility and fast degradation rates of a few weeks in vivo, and were reviewed by Bettinger^[^ ^36^ ^]^ or Chen et al.^[^ ^32^ ^]^ PPSs are synthesized through a polycondensation reaction of polyols (most commonly food additives such as glycerol, xylitol, or sorbitol) with sebacic acid, which is industrially derived from castor oil. Poly(glycerol sebacate) (PGS) offers mechanical properties suitable for soft robotics with a Young's modulus ranging from 0.05 to 1.5 MPa and high reversible stretchability of up to 500% strain.^[^ ^37^ ^]^ The mechanical properties are mainly influenced by synthesis time and temperature during the polycondensation reaction. Despite the need for synthesis, the use of bioderived/food grade materials render PGS a low‐cost material approach. Yet, PPSs require to be molded directly after synthesis, which limits their processability to sheets, foams, and simple 3D structures. The limited processability may hamper its widespread market introduction and renders their employment in soft robotics challenging. However, first biodegradable soft robots were assembled from PGS sheets with cyanoacrylate as adhesive (see Section 6.2).^[^ ^38^ ^]^ Rueben et al. added itaconic acid during the synthesis process to initiate the crosslinking reaction of PGS via photo crosslinking and enable an easier adaption of PGS in soft robotics labs.^[^ ^39^ ^]^ Poly(glycerol sebacate itaconate) (PGSI) crosslinks under illumination with 250 nm UV light and results in a soft (Young's modulus, 130–200 kPa) material with moderate stretchability up to 140% strain. If not used as the main material, PGS may be useful as an elastic coating for biodegradable devices and soft robots that operate in wet environments.^[^ ^40^ ^]^ Hydrophobic in nature, PGS barely swells in water (<5%) yet can be applied through a spray coating technique while maintaining a high stretchability of >200% strain.

Poly(diol citrates) (PDCs) represent another biocompatible family of synthetic biodegradable elastomers. Similar to PPSs, PDCs are synthesized through a thermal polycondensation reaction of citric acid with aliphatic diols, usually 1,8‐octanediol.^[^ ^41^ ^]^ Unlike PGS that requires synthesis temperatures above 120 °C, poly(1,8‐octanediol citrate) (POC) polymerizes at temperatures below 80 °C.^[^ ^42^ ^]^ Adjusting the monomer ratios, postpolymerization temperature and time define both degradation rates and mechanical properties. POC degrades within 6 months under physiological conditions (PBS, 37 °C), however, faster rates (2 months) were observed in vivo when catalyzed by enzymes. The mechanical properties of POC fall into the desired range for soft robots with a Young's modulus ranging from 0.9 MPa to 16 MPa and an ultimate strain of up to 265%. As an improvement, Yang and co‐workers introduced a dual crosslinking mechanism for POC by further adding maleic anhydride to obtain poly(octamethylene maleate (anhydride) citrate) (POMaC), which allows crosslinking via UV illumination and/or polycondensation.^[^ ^43^ ^]^ Favorably, they achieved softer formulations (Young's modulus from 0.04 to 1.5 MPa) compared to POC and high ultimate strain from 50% to 500 %. However, the highest stretchability corresponds to the formulation with the lowest modulus and vice versa. The easy synthesis process, its low‐cost monomers, and tunable degradation behavior makes POMaC a substrate candidate for implantable electronics (see Section 4.1). Additionally, POMaC was used for stretchable strain and pressure sensors.^[^ ^44^ ^]^ Although this material has good mechanical properties, it is yet unclear if POMaC will find use for biodegradable or bioresorbable soft robotics. Unlike PGS, the polymer network of POMaC is hydrophilic and heavily swells in water. This is not a disadvantage per se, as water swollen polymers (hydrogels) are an employed material class in soft robotics. However it makes POMaC a direct competitor to bioderived hydrogels that show similar properties but do not necessarily require synthesis.

Bioderived hydrogels that are also biodegradable are a promising material class for sustainable soft robotics. Hydrogels very closely resemble the mechanical structure of the human body—we are in essence a water swollen polymer ourselves,— allow all kinds of water‐soluble additives, and exhibit excellent biocompatibility. They cover a wide range of mechanics, from extremely soft to tough, mimicking brain tissues, muscles, or tendons. Bioderived building blocks such as alginate, starch, or gelatin are inexpensive renewable resources, widely (commercially) available, and biodegradable, yet they often fail to provide sufficient mechanical integrity under high loads, which are required for soft robotics. However, various strategies exist to boost the performance of such materials and are reviewed in the last part of this section.

Alginate‐based hydrogels are widely established in biomedical applications including tissue engineering, drug delivery, and wound care. Their successful implementation in those fields mainly stems from their biocompatibility and the versatile range of crosslinking strategies to produce gels.^[^ ^45^ ^]^ Typically, alginates form ionic bonds when crosslinked in ionic solutions (e.g., Ca^2+^), however they also can be chemically crosslinked initiated by UV‐irradiation or temperature.^[^ ^46^, ^47^ ^]^ The ability to reversibly form ionic bonds has attracted much attention for technological used double network hydrogels that benefit from alginate as dissipating conetwork. Polyacrylamide/alginate hydrogels were stretched to >1000% linear strain, even when notched.^[^ ^48^ ^]^ The alginate interpenetrating network dissipates energy due to the breaking and reforming of its ionic bonds, while the polyacrylamide network stabilizes the hydrogel, and therefore renders hydrogels with excellent fracture toughness. While alginate shows only moderate mechanical performance on its own (Young's modulus < 100 kPa, ultimate strain <100%), this strategy can be a frugal approach in combination with biodegradable polymer matrices instead of polyacrylamide. Zhu et al. reported a double network hydrogel consisting of chemically crosslinked hyaluronic acid (HA) and ionically crosslinked sodium alginate as dissipating interpenetrating network.^[^ ^49^ ^]^ With this approach they were able to increase the ultimate strain of HA hydrogels from 200% to above 400%, yet for a low Young's modulus of 0.9 kPa.

In the group of polysaccharides, biodegradable elastomers based on plasticized starch—also called thermoplastic starch (TPS)—are reported. TPS, which is mainly employed as bioplastic for ecofriendly food packaging, is based on disrupted native starch with glycerol (or other polyols) as plasticizer and has moderate mechanical properties when used as elastomer (ultimate strain <100 %).^[^ ^50^, ^51^ ^]^ Yet, it is used as filler material in silicone elastomers or as chain extender for polyurethanes to increase otherwise very slow degradation rates. With an increasing starch content, the mechanical properties of the elastomers rapidly decrease. Ceseracciu et al. optimized starch/polydimethylsiloxane composites to achieve ultimate strain values above 500% while featuring 30 wt% biomass content.^[^ ^52^ ^]^ The degradation rates, however, remain low with 6 years estimated degradation time in seawater.

Similar to polysaccharide‐based hydrogels, gelatin‐based gels offer a wide range of tunable mechanics, low material costs, and biodegradation without the need of industrial composting processes. Gelatin is one of the most successful biopolymers, commercially available, and has various applications across many sectors from food industry to medicine. The simplest way to achieve gelatin gels is to mix gelatin powder in hot water. The gelation process sets in under cooling, when the sol‐gel transition temperature is reached. During the gelation of gelatin, the individual polymer strands form triple helices and physical crosslinks, building the biopolymer network. The resulting gel however, easily breaks upon stretching and rapidly dries in ambient conditions—like most hydrogels—due to evaporation of water, which results in material stiffening and shrinking. Several strategies exist that address these issues from introducing additional additives, salt solution treatment, to covalent crosslinking. The synthesis of methacrylated gelatin (GelMa) renders a prepolymer solution that is covalently crosslinked under UV illumination. This gives a technological advantage as the gel forming process is now precisely controlled by adjusting the photoinitiator concentration or light intensity and enables 3D‐printing of GelMa with commercially available bioprinters.^[^ ^53^, ^54^ ^]^ Photo‐crosslinked GelMa gels exhibit a tunable Young's modulus in the range of 0.1 to 0.4 MPa and an ultimate strain of up to 100%.^[^ ^55^ ^]^ Its tunable mechanics and inherent bioactivity—biodegradation within 3 weeks in collagenase at 37 °C were reported^[^ ^56^ ^]^—make GelMa a versatile material choice for biomedical applications including tissue engineering. For soft robotics, the mechanical properties of GelMa require additional improvements towards higher stretchability.

Wu et al. resolved this issue by photo‐initiated polymerization of hydrogen bonding 2‐vinyl‐4,6‐diamino‐1,3,5‐triazine (VDT) and GelMa.^[^ ^57^ ^]^ They achieved a pH responsive biodegradable hydrogel with high stretchability and tunable Young's modulus in the range of 0.4 to 2 MPa. Compared to GelMa, the ultimate strain could be increased from ≈100% to over 400% for the best PVDT‐GelMa formulations. In acidic solutions (pH 1.2) the hydrogen bonds of the network break up leading to a reduction of the materials Young's modulus. The covalent crosslinks keep the hydrogel in shape, yet they degrade after 48 h in gastric fluids. The authors report no harmful residues during the degradation process and envision drug delivery applications. For soft robotic applications, the improvements in mechanics result in higher material costs and the need for synthesis. Additionally, while these hydrogels reach an equilibrium swelling point in pH neutral water (equilibrium water content, 60–70 wt%), the authors do not discuss stability of gels under ambient conditions. This however is a necessary requirement for robots operated in air.

A frugal approach is to introduce (nonvolatile) plasticizers such as glycerol (or other polyols) as cosolvents in gelatin gels. After evaporation of free water, the gels remain stretchable due to the nonvolatile cosolvent. Shintake et al. used this concept to fabricate gelatin‐based gels with a Young's modulus of 0.7–2.7 MPa and an ultimate strain of ≈150%.^[^ ^58^ ^]^ Gravimetric stability tests under ambient conditions show an initial mass loss of 70 wt% within the first 24 h due to the loss of water but then a constant mass for at least one week. How the mechanical properties change during this period was not investigated, however large volume changes after curing affect the material processability in general. Nevertheless, the authors demonstrated a pneumatic soft actuator capable of repeated actuation in the order of 10 cycles. Aiming for higher durability, ultimate strains beyond 200% are required to reduce possible failures at high actuation strains.

An efficient strategy to increase the mechanical properties of gelatin gels is to soak them in highly concentrated salt solutions. He et al. reported a one‐step soaking process in ammonium sulfate ((NH_4_)_2_SO_4_) for cured gelatin gels, which dehydrates the gels as a result of a “salting out” effect.^[^ ^59^ ^]^ Higher concentrations of NH_4_^+^ and SO_4_^2−^ ions—both strongly kosmotropic ions in the Hofmeister series—increase biopolymer–biopolymer interactions and reduce the solubility of gelatin in water. As a consequence, the gelatin gels shrink in ammonium sulfate, with strongly enhanced mechanical properties. The best gels reported by He et al. achieved an increase in ultimate strain from 90% to over 500% by soaking in 25 wt% ammonium sulfate solution. Likewise, the Young's modulus and ultimate stress both increased to 0.6 and 3.2 MPa, respectively. This method is universally applicable for gelatin gels, but large volume changes due to the salt treatment must be accounted for. Qin et al. achieved similar results by soaking gelatin gels in 20 wt% Na_3_/C_6_H_8_O_7_ solution.^[^ ^60^ ^]^ In their report, they demonstrated that the high salt content further reduces evaporation of water in ambient conditions, with limited water (mass) loss (≈9 %) after one week.

So far, strategies based on photo‐crosslinking, inclusion of additional monomers or plasticizers, or salt treatment help to enhance the mechanical properties of gelatin gels, but on the cost of processability, degradability, or price. Baumgartner and co‐workers fabricated a biodegradable gelatin‐based biogel from food save constituents with increased performance, low fabrication costs at ambient conditions, and long‐term shape and property stability.^[^ ^8^ ^]^ The biogels are stretchable to above 320% ultimate strain and have tunable mechanics covering a Young's modulus range from 0.03 to 3 MPa. The boost in stretchability is achieved by utilizing sugar syrup as cosolvent that contains a high amount of maltose and related sugars. These sugars enhance helix–helix association in the biogel, which leads to higher stretchability. Glycerol as a second cosolvent prevents evaporation of water stored in the polymer network and leads to volume stability of 95% directly after casting. Tuning the water glycerol ratio of the gels also resulted in constant mechanical properties for more than a year. Citric acid as an additive prevents microbial growth and degradation when stored under ambient conditions, yet the biogels degrade within 10 d in wastewater. While the biogel can be worn on‐skin for prolonged times, the authors note that in vivo applications might require additional coatings to increase its lifetime. The increased mechanical strength, good processability, and long‐term stability enable durable soft pneumatic actuators and soft e‐skins, which are worn on‐skin without the need for additional adhesives.

### 3.3 Biodegradable Plastics and Polysaccharides

Biodegradable plastics (Bioplastics) offer a structural support for soft robots from rigid parts to thin lightweight films. For the market, bioplastics are increasingly employed as packaging material or as replacement for conventional single use plastics, governed by recent regulations of the European Union or China. Biodegradable thermoplastics or thermosets are the prevalent materials since they are industrially processable by injection molding, drawing, or—more important for soft robotic labs— 3D‐printing. Although bioplastics are biodegradable, they are fabricated from fossil fuel, biomass, or renewable resources (including composites). In this section, we review selected biodegradable (thermoplastic) plastics from nonrenewable and renewable sources and discuss their mechanics and potential use for soft robotics. More detailed information on bioplastics in general are found in recent reviews.^[^ ^61^, ^62^ ^]^

**Figure 4:**
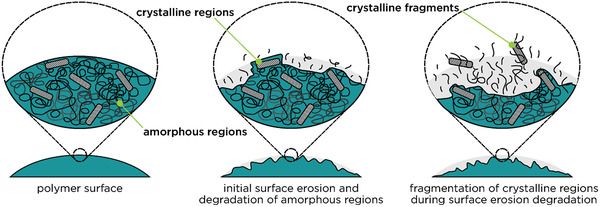
Hydrolytic surface erosion (of PCL). The polymer degrades by a process that targets the surface where amorphous regions dissociate first, followed by the removal of crystalline fragments. Adapted with permission.^[^ ^65^ ^]^ Copyright 2010, Elsevier.

Similar to synthetic biodegradable elastomers, polyesters are commonly used for bioplastics. Poly(ε‐caprolactone) (PCL) is a semicrystalline synthetic polyester, completely derived from fossil fuel, with good processability and a low melting point of 60 °C. The mechanical properties of PCL mainly depend on its molecular weight, crystallinity, as well as porosity, and range from 250 to 400 MPa Young's modulus.^[^ ^63^, ^64^ ^]^ The (one order of magnitude) lower Young's modulus compared to conventional plastics (PET, polyethylene (PE), acrylonitrile butadiene styrene (ABS) etc.) is common for bioplastics, as synthetic or bioderived plastics offer a wider range of mechanical properties. Their stretchability, however, is typically limited to 5–10% strain until their yield point. PCL is stretchable up to 20% strain before irreversible plastic deformations occur.^[^ ^63^ ^]^ Compared to the human body, PCL has similar properties to tendons, yet a lower tensile strength. The typical degradation process of PCL includes hydrolytic surface erosion due to the cleavage over ester bonds in the presence of moisture and high temperatures^[^ ^65^ ^]^ (Figure 2b, **Figure** **4**). Additionally, the degradation process is accelerated by enzymatic degradation of natural organisms.^[^ ^61^ ^]^ Hosni et al. studied the degradation of PCL under different temperature conditions in soil and compost showing a complete degradation of PCL within 100 d at 50 °C.^[^ ^66^ ^]^ Under physiological conditions however, PCL barely degrades due to the lack of suitable enzymes resulting in degradation times in the range of 2–4 years,^[^ ^67^ ^]^ and similar timescales for degradation in seawater.^[^ ^22^ ^]^

In contrast to fossil fuel‐based plastics, bioplastics that are derived from renewable resources are more desirable for sustainable technologies. Polyhydroxyalkanoates (PHAs) are a class of biocompatible and biodegradable thermoplastics that are synthesized from bacteria involved in the fermentation of sugars or vegetable oils. They offer a broad variety of monomers with (conventional) plastic like properties and degrade into nontoxic components, rendering PHAs suitable for biomedical applications. With a melting point of typically 170 °C, these polymers are compatible with standard polymer processing such as solution casting or melt extrusion. Compared to PCL, the stiffer PHAs such as polyhydroxybutyrate (PHB) or poly‐3‐hydroxybutyrate (P3HB) offer a higher Young's modulus in the range of 1.5 to 2.5 GPa, more similar to commodity plastics.^[^ ^64^, ^68^ ^]^ In general, the mechanical properties of PHAs offer a wide range and are modulated by the length of the side chains—short side chains render hard crystalline materials, long side chains render elastomeric materials—and by the distance between ester linkages in the polymer backbone.^[^ ^41^ ^]^ The degradation of PHAs occurs similar to PCL with a partial hydrolytic degradation and further enzymatic cleavage of the polymer. The microorganisms that secrete suitable enzymes mostly occur in soil and compost, but only few in maritime environments, leading to degradation times of several weeks in compost and few years in seawater.^[^ ^22^, ^69^ ^]^ However, the degradation times depend on many factors including crystallinity, plasticizers, temperature, available surface, and available enzymes.

In line of biodegradable synthetic plastics, polylactide (PLA) is an aliphatic polyester derived from renewable resources such as corn starch. It is one of the most important industrially used bioplastics due to its excellent processability and the most common filament type in 3D printing. Typically, PLA has a melting point between 130 °C and 180 °C, good adhesion to various surfaces when printed, and negligible material shrinkage, which makes it an ideal 3D‐printing material for many applications. Its mechanical properties are similar to conventional plastics with a Young's modulus of 2–3.5 GPa and an ultimate strain of 4–7%.^[^ ^70^, ^71^ ^]^ Yet, with a glass transition temperature of ≈60 °C PLA has only moderate temperature resilience, hampering a more widespread use comparable to commodity plastics. The chiral structure of lactide acid (l,l‐lactide and d‐lactide) provides related polymers poly(l‐lactide) (PLLA) and poly(d‐lactide) (PDLA), plus various combinations such as poly(d,l‐lactide) (PDLLA). Both PLLA and PDLA form complexes with higher crystallinity and hence higher Young's modulus of 4 GPa as well as slower degradation.^[^ ^71^ ^]^ The degradation of PLA in general mainly depends on temperature and available enzymes. Degradation in industrial composting facilities, which operate near the glass transition temperature of PLA, can be completed in less than 90 d,^[^ ^62^ ^]^ while it shows almost no reasonable signs of degradation in seawater.^[^ ^22^ ^]^ Plasticizers or copolymers can have favorable effects toward shorter degradation times. Poly(lactic‐*co*‐glycolic) acid (PLGA), for example, offers tunable degradation rates through adjusting the ratio of lactic and glycolic acid, which degrades 13 times faster.^[^ ^72^ ^]^ Even in seawater, PLGA degrades completely within 2 years.^[^ ^22^ ^]^

Addressing disadvantages emerging from low glass transition temperatures (*T*_g_), Park and co‐workers recently developed a high‐performance thermoplastic polymer from (partially) renewable resources with a *T*_g_ of 212 °C.^[^ ^73^ ^]^ In analogy to plastics containing toxic bisphenol‐A, they alternatively incorporate bio‐derived isosorbide (1,4:3,6‐dianhydro‐d‐glucitol, ISB) into fossil‐based poly(arylene ether)s (PAEs) with a high molecular weight of over 100 kg mol^−1^. With a low thermal expansion coefficient of 23.8 and 81.2 ppm K^−1^, Park et al. envision their material as suitable replacement for plastics in printed circuit boards (PCBs). Additionally, to the good thermal resilience, it offers a high Young's modulus of 3.7 GPa and an ultimate tensile strength of 78 MPa at an ultimate strain of almost 8%. Although the ISB‐PAE is not a biodegradable thermoplastic, the authors demonstrate an injection molding compatible recycling process to address sustainability issues. The authors also note, that replacing PAE with bioderived plastics will be required to increase the environmental friendliness of the material.

Plastic films made from polysaccharides such as chitin, cellulose, or starch are a sustainable material approach from renewable resources with faster degradation rates compared to synthetic bioplastics. The faster degradation mainly stems from the highly hydrophilic nature of polysaccharides, which makes their material properties sensitive to humid environments. The low gas permeability of starch blends led to market introduction as food packaging material (however often in combination with synthetic bioplastics) and has been reviewed.^[^ ^74^, ^75^ ^]^ Their excellent biocompatibility, nontoxic biodegradation residues, and largely available resources render polysaccharides a frugal material class for robots made of paper or cotton yarns, and with improvements concerning their mechanical properties, plastic‐like films, e.g., as substrate for electronics are feasible. Films based on thermoplastic starch/chitosan blends have a Young's modulus in the range of 1–1.2 GPa with a tensile strength of about 12 MPa and an improved water vapor permeability.^[^ ^76^ ^]^ Softer films are typically fabricated by introducing plasticizers such as glycerol or sorbitol, which also extends their ultimate strain (yet at a low level).^[^ ^77^ ^]^ Blends of starch with biodegradable polyesters (PCL, PHA, or PLA) usually have a lower water permeability than pure thermoplastic starch films and faster degradation time than synthetic bioplastics, which makes them a good compromise for industrial applications.^[^ ^78^ ^]^ Besides starch and chitin, cellulose— the prevalent polysaccharide—is extensively researched due to various fiber types including cellulose nanofibrils (CNF) and crystals (CNC), with applications in construction, biomedicine, and electronics.^[^ ^79^, ^80^ ^]^ The high Young's modulus in the range of 10–15 GPa makes CNF composites a strong structural support material and a feasible strategy as reinforcement for various other (bio‐)polymers.^[^ ^80^ ^]^

### 3.4 Living Materials

A conceptional completely different approach, yet highly challenging, is the use of self‐growing materials for robotics. The main idea is that materials are not assembled but grown into the desired shape. This is achievable by either using materials with programmed volume expansion, such as ontogenetically modified yeast,^[^ ^81^ ^]^ or living materials such as tissues, plants, or fungus. In addition, robots that mimic biological growth by the incremental addition of material and could lead to systems that adapt, change, and evolve during operation.^[^ ^82^ ^]^ Imitating the growth of plant roots, Sadeghi and co‐workers developed a customized PLA‐filament 3D printer, which is part of the (growing) robotic body and progresses through soil to build root‐like structures.^[^ ^83^ ^]^ The decisions on the growing directions are made autonomously by sensing, e.g., humidity gradients in the earth. Additive manufacturing (like 3D printing) in essence is like growing plants, however the growing process is technologically achieved and not intrinsic to the used materials. Self‐growing materials like cells, or fungus mycelium offer great potential as they can grow through various scaffolds to build robotic parts and materials with new functionalities. At the same time such materials are intrinsically biodegradable, renewable, and require only small amounts of energy to grow. However, self‐growing materials are rather unexplored for robotics. While cells, for example, are mainly studied for tissue engineering applications, their utilization for small scale soft robotic actuators is still in its infancy, as reviewed by Ricotti et al.^[^ ^84^ ^]^ Regenerative medicine and soft robotics equally benefit from research efforts toward self‐growing actuators and materials. In this section, we exploit self‐growing materials, which provide interesting properties for sustainable soft and lightweight robotics.

Mycelium composites are an emerging class of low‐cost biomaterials, which recently find application as sustainable material technology for acoustic damping, insulation panels, or packaging.^[^ ^85^ ^]^ Mycelium—the collective of hyphen networks, which builds the main body of fungus—naturally grows into different abundant agricultural by‐products and wastes, and renders composites that provide structural integrity at very low densities. In the manufacturing process of mycelium composites, fibrous substrates, typically straw or sawdust, are upcycled to keep the fabrication costs low and allow for closed product life cycles. However, any type of substrate—as long as it supports fungal growth—can be used for composites and mainly defines its (mechanical) properties. This allows for an easy way to reshape materials, as the loose substrates can be filled into nondigestible (metal, plastic, or glass) molds, resulting in a mycelium composite of the respective shape. The growth takes place at ambient conditions with no force exerted on the mold, which also allows filigree mold structures.

Mechanically robust composites with a Young's modulus of 20 MPa and an ultimate tensile strain of 10% are achieved by the growth of *Pleurotus ostreatus* fungus in cellulose‐potato dextrose.^[^ ^86^ ^]^ The foam‐like structure of mycelium composites naturally results in lower mechanical strength when compared to solid plastics, however they also exhibit considerably lower densities in the range of 100–300 kg m^−3^.^[^ ^87^ ^]^ Cold‐ or hot‐pressing of mycelium panels are strategies to decrease the materials porosity and to increase the Young's modulus—up to an order of magnitude—when stiffer material properties are required.^[^ ^88^ ^]^ While most of the mycelium forms highly porous networks, the surface of mycelium composites is built from a dense hyphen layer, which results in unique material properties. The surface is highly hydrophobic showing contact angles of 120° with water,^[^ ^86^ ^]^ which prevents swelling of the material in a humid environment and provides opportunities for electronics fabricated on mycelium as substrate. The porous network in contrast is highly hydrophilic, able to absorb large amounts of water when immersed. These antagonistic properties stem from the hyphen cell wall architecture that mainly consists of the polysaccharides chitin and glucan but also features a protein layer. While the hydrophobic protein layer densely covers mycelium composites, the porous inside contains hydrophilic polysaccharides from the mycelium and the substrate.

In contrast to mycelium, which serves as structural support material on large scale, muscle tissues can be utilized for dynamic biohybrid actuators for highly miniaturized robots. Biohybrid actuators combine biological actuation (from extracted or grown motile cells or muscle tissue) with small artificial machines in the milli‐ and microscale. Although the fabrication and control of biohybrid actuators is highly challenging, demonstrations of biohybrid robots—from manipulators^[^ ^11^ ^]^ to color changing structures^[^ ^89^ ^]^—show promising results, which are discussed in more detail in Section 6.4. Typically, scalable or general purpose biohybrid actuators are based on (optogenetically modified) cardiomyocytes, insect‐self contractile tissues, or engineered skeletal muscle tissues and recently have been reviewed in detail.^[^ ^84^ ^]^ Cardiomyocytes are striated muscle cells that contract as a collective when (electrically) stimulated. As their fabrication involves tissue engineering methods, the emerging interest in biohybrid actuators extended the field of robotics to research activities that normally focus on regenerative medicine. Stimulation of cardiomyocytes can be achieved by integrating carbon nanotubes in hydrogels that serve as both substrate and electrodes for the cells.^[^ ^90^ ^]^ However, the achieved forces and contractions are very low compared to cardiac muscle tissue. Additionally, cardiomyocytes spontaneously self‐contract, which makes them an unfavorable solution for most robotic applications. Optogenetic modifications provide increased control over these cells (although not eliminating spontaneous contraction), as they artificially employ light responsiveness, which allows for external control of contraction frequency.^[^ ^91^ ^]^ Besides control issues, cardiac muscle tissue has reduced stability at room temperature, with lifetimes of typically one week.^[^ ^92^ ^]^ Insect cells/tissues are a useful alternative compared to mammalian tissues as they provide longer lifetimes (up to 90 d) and robustness to large temperature, pH, or osmotic pressure changes.^[^ ^93^ ^]^ Still, controlling such cells remains highly challenging and—although systematic analysis of various stimulation approaches—remains the main research aim to be addressed. Recent advances that allow for an on–off controllability and modular designs were achieved with skeletal muscle tissues. Skeletal muscles are composed of aligned bundles of myofibrils (myoblasts fused through myogenesis) and controlled by the somatic nervous system. Replicating contractile fibers from a single myoblast is a challenging task, which involves tissue engineering methods. Yet, skeletal muscle tissue was successfully utilized as actuators in robotic applications, where myoblast‐laden hydrogel sheets were grown into the robotic skeleton.^[^ ^11^ ^]^ The progress within biohybrid actuators established this field as an exciting branch of bio‐inspired robotics with great potential towards highly miniaturized robots. Even if sustainability issues are hard to address with this technology—low‐cost, largescale production of engineered muscle tissue is still elusive—it fundamentally changes our perception of robots, as with the increasing incorporation of “living” materials, our robots change from purely technology‐based towards a novel life form.

### 3.5 Biodegradable Metals

The very opposite of soft living tissues are metals. Lying on the far end of the Young's modulus spectrum, this material class mainly builds the electronic circuitry for robots and is essential for more complex autonomous operation. Metallic conductors—such as gold or copper, which are typically employed in electronic circuit boards—are rather resilient against corrosion (noble metals), or degrade into nonbiocompatible residues (in the case of copper). Biodegradable metals are a subclass of metals that completely disintegrate into biocompatible building blocks, spontaneously or through a trigger and in suitable environments. This includes metals that completely dissolve in biofluids, also called bioresorbable or bioabsorbable metals, which are in high demand for electronic medical implants for diagnostics or therapeutics (recently reviewed by Choi et al.^[^ ^94^ ^]^), but also for transient robots operating in vivo. In this section, we briefly discuss the degradation of common biodegradable metals as a sustainable material approach for robotics, comprehensive reviews on transient and biodegradable metals are found here.^[^ ^95^, ^96^ ^]^

An obvious choice for biodegradable metals is those, which are also essential nutrients. Calcium (Ca), magnesium (Mg), iron (Fe), zinc (Zn), and manganese (Mn) are typical metals in our body and (in small amounts) part of our diet. Others, frequently used in bioresorbable electronics, are tungsten (W) and molybdenum (Mo). Mg and its alloys are an employed material solution for various implantable electronic devices. With a daily allowance of 0.7 g, reasonable amounts of Mg are safely absorbed by the body and serve there as a promoter for muscle contraction. It offers fast degradation rates of a few micrometer per day in water or biofluids and safe degradation products through the hydrolysis of Mg: Mg + 2H_2_O → Mg(OH)_2_ + H_2_.^[^ ^95^ ^]^ While enzymes play a minor role in the degradation of metals (hydrolysis is predominant) the degradation kinetics mainly depend on temperature, pH, and salts present in the respective solutions. Cl^−^ ions are greatly promoting corrosion of metals, leading to short lifetimes when used in maritime environments.^[^ ^97^ ^]^ The pH (at least in the range of pH 5–8) has a minor effect on Mg or the Mg–Zn alloy (AZ91D).^[^ ^98^ ^]^ Mo, however, shows a strong dependence on oxygen solubility and hence degrades faster in solutions with low pH.^[^ ^95^ ^]^ How the rates change for natural (partially humid) environments, such as soil or compost has not been investigated (to our knowledge) so far. However, it might be in the range of degradation rates in DI‐water depending on precipitation and soil composition. For more complex assemblies of electronics that include metals, semiconductors, and plastics, the degradation rates will further largely depend on the device design and degradability of the individual components. Approaches toward fully and partially degradable sustainable electronics are reviewed in the next section.

## 4 Electronics

Truly autonomous (soft or rigid) robots require at least basic electronics to sense and interact with their surroundings. To date, most soft machines compensate for these challenges through mechanical design concepts and material choices, which offload some control tasks to the soft structure of their body. This key advantage immensely reduces the need of sensors, feedback loops, and real‐time computation and inspired the research field of morphological computation. However, with the growing complexity in the tasks of autonomous robots, sensor networks become indispensable. Even relatively simple animals such as worms require mechanical feedback, making pressure and strain gauges one of the first elemental sensors. The human skin additionally provides temperature and humidity sensing, and an octopus uses distributed light‐sensitive cells to actively camouflage through mimicking its environment. Addressing and processing this information requires transistors, memory, and communication tools, along with sufficient power supplies to enable actuation and process information. Here, selecting the right amount of responsiveness is an efficient and sustainable way to address certain tasks and save computational effort and energy.

In addition to the scientific challenges, targeting integration and performance, economic and environmental concerns to lower both production costs and material waste are imminent^[^ ^99^ ^]^ for the robotic body and its soft sensor skin. With a growing number of different devices, emerging in both the medical and consumer electronics sector, designs, fabrication steps, and products quickly alter, which requires adaptable fabrication lines and rigorous waste management. These concerns are likewise addressed by the community around flexible, printed and stretchable electronics, where the progress in processing techniques and materials already enabled sophisticated e‐skins with multimodal sensing capabilities, mainly for biomedical applications.^[^ ^100^ ^]^ The latter is also the main driving force behind biocompatible, nontoxic, and environmentally friendly electronics.^[^ ^3^, ^101^, ^102^ ^]^ With the rise of bioresorbable or transient electronics, which started with a few ground‐breaking developments,^[^ ^103^, ^104^, ^105^ ^]^ new classes of materials, composites, and fabrication techniques evolved with benefits for users, producers, and the environment. Key technological advances were recently reviewed, highlighting their importance for bioresorbable implants.^[^ ^12^, ^106^ ^]^ However, extending these achievements to consumer electronics or robotics, will require additional academic and translational efforts. Yet, sustainable soft robotics can greatly benefit from these achievements. Joining these fields seems like a meant‐to‐be match and naturally provides a fertile platform to learn from each other and advance frugally. Here, we highlight materials and design concepts of sensors, switching elements, memory, and communication tools, such as light emitting diodes (LEDs), toward first proof of principle degradable e‐skins that are promising for adaption in soft robotics.

### 4.1 Input: Sensors

The perception of touch is one of the most essential feedbacks for robotics (and humans) to manipulate objects and interact with their environment. Nature provided us a skin with extremely sensitive sensor networks that respond to a variety of stimuli, from small vibrations and flow of air to stretches, touch, or heavy impacts.^[^ ^107^ ^]^ Replicating and understanding the complexity of human skin from an electronics and materials engineering point of view is one of the major challenges in the field.^[^ ^44^, ^108^ ^]^ A robotic artificial skin will differentiate external stimuli with a network of sensors, which are sensitive to pressure, strain, vibrations, and (air) flow.

Typical pressure sensors, even stretchable ones, can be categorized into piezoelectric (resistive or capacitive), capacitive, or resistive sensors. Independent of the sensing method and actual design, each pressure sensor requires two electrodes (bottom and top), an intermediate layer that allows deformation, and (if required) a substrate or encapsulation. Truly sustainable electronics require solutions for each part of the sensor, the integration level of ecofriendly materials however broadly varies.

**Figure 5:**
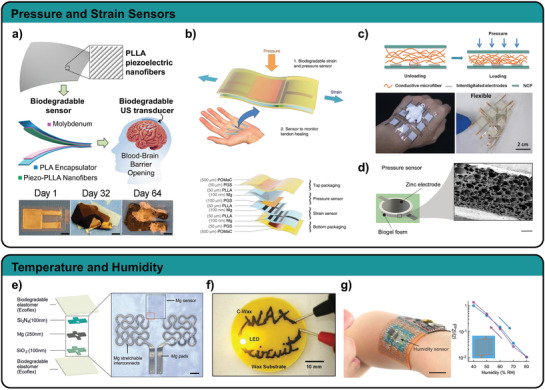
Biodegradable pressure, strain, temperature, and humidity sensors. a) A piezoelectric pressure sensor based on oriented PLLA nanofibers. Reproduced with permission.^[^ ^111^ ^]^ Copyright 2020, National Academy of Sciences. b) A multilayer design allows independent pressure and strain measurements with completely bioresorbable materials. Reproduced with permission.^[^ ^44^ ^]^ Copyright 2018, The Authors, published by Springer Nature. c) Cellulose nanofibers are used as deformable dielectric in a capacitive pressure sensor matrix. Reproduced with permission.^[^ ^109^ ^]^ Copyright 2019, American Chemical Society. d) Capacitive pressure sensor based on a biogel foam and Zn electrodes. Reproduced with permission.^[^ ^8^ ^]^ Copyright 2020, Springer Nature. e) Stretchable temperature sensor utilizing Mg serpentines and Ecoflex as encapsulation. Reproduced with permission.^[^ ^118^ ^]^ Copyright 2017, Wiley. f) A wax structure mixed with W particles is used as conductor and to measure temperature. Reproduced with permission.^[^ ^119^ ^]^ Copyright 2018, Wiley. g) Humidity sensor based on interdigitated Zn electrodes shows a response of two orders of magnitude. Reproduced with permission.^[^ ^8^ ^]^ Copyright 2020, Springer Nature.

Gao et al. fabricated an all paper‐based piezoresistive pressure sensor that uses Ag nanowires (NWs) as electrodes.^[^ ^109^ ^]^ Nanocellulose paper (with a thickness <500 µm) was used as substrate and encapsulation for the sensor due to its transparency and smooth surface. A printed dispersion of Ag NWs forms interdigitated electrodes, which are covered with Ag NWs loaded tissue paper (as intermediate layer). Applying pressure on the sensor increases the contact area of the conductive tissue fibers and decreases the resistance between the electrodes (**Figure** **5**c). The sensor shows good stability under repeated loading and folding, and high sensitivity of 1.5 kPa^−1^ (relative current change when measured at a constant potential) in the range between 30 Pa and 30 kPa. Progressing completely biodegradable sensors would require replacing the Ag NWs with biodegradable metals. This, however, is challenging due to the high surface/volume ratio of NWs and therefore fast degradation under humid conditions.

A completely biodegradable device envisioned for implants uses a multilayer approach with Mo as electrodes, PLLA as intermediate layer, and PLA as encapsulation.^[^ ^110^ ^]^ The authors modified the crystallinity and orientation degree of the PLLA polymer chains to make it piezoelectric. Therefore, they annealed a PLLA film in stretched state to modify the crystallinity, followed by a 45° cut along the stretching direction. With the sensor the authors could reliably measure vibrations and pressure with a sensitivity of 0.06 kPa^−1^ (relative voltage change) in the range between 2 and 18 kPa and a much higher sensitivity up to 2 kPa pressure input. Improving their concept, Curry and co‐workers utilized electrospun PLLA fibers instead of PLLA sheets, increasing the sensitivity by a factor of 1.8.^[^ ^111^ ^]^ They implanted their sensor in a mouse model to monitor physiological pressures and second used it as a transducer to stimulate opening of the brain blood barrier (Figure 5a). Although the authors state that their devices safely degrade under physiological conditions, the degradation rates are slow. Accelerated degradation at 70 °C in phosphate buffered saline (PBS) only achieved partial degradation of their device after 64 d, due to the slow degradation rates of PLA and Mo.

Khalid et al. achieved faster degradation in PBS at room temperature using a thin (110 nm) Fe/Zn bilayer structure as electrodes, electrospun PLGA/PCL fibers as dielectric layer and polyvinyl alcohol (PVA) sheets as encapsulation.^[^ ^112^ ^]^ Their capacitive sensor shows a similar performance with a sensitivity of 0.06 kPa^−1^ in the range of 2–5 kPa (relative capacity change) but partially degrades within 18 d. The pressure sensors so far are flexible thin films that do not require any stretchability. Highly conformable and stretchable forms of pressure sensors—in this case such sensors are often susceptible to pressure and strain—are necessary for soft robotics or wearable e‐skins.

Stretchable electrodes are achieved by loading silk fibroin sheets with Ag NWs.^[^ ^113^ ^]^ Using Ecoflex as a dielectric layer results in a stretchable capacitive sensor that responds to pressure and strain. The sensor shows a high sensitivity of 1.8 × 10^−2^ (relative capacity change) at low pressures up to 20 kPa and a lower sensitivity of 5.5 × 10^−4^ in a broad pressure range between 200 and 700 kPa. Equally the relative capacity changes by 0.38 per tensile strain. Similar to other sensors, the used materials are only partially degradable or have low degradation rates (Ecoflex for example) under natural conditions. Differentiating pressure from strain is additionally required for most soft robotic or electronic applications.

A purely strain responsive sensor was fabricated by Wang and co‐workers from carbonized silk fabric.^[^ ^114^ ^]^ Therefore, plain‐wave silk fabric was carbonized at 950 °C in argon/hydrogen gas and subsequently encapsulated in Ecoflex. Such carbon/elastomer composites typically have a high (nonlinear) resistance increase upon deformation, resulting in a gauge factor (relative resistance change per strain) in the range of 6–10 up to a strain of 200%. The degradation behavior of this device was not investigated but is dominated by the rates of the encapsulation (in this case Ecoflex). Using bioderived encapsulations with shorter and tunable degradation profiles will further increase the ecofriendliness of these devices.

Most commonly, pressure sensors use bulk dielectrics or porous structures, obtained either by electrospinning or by using foams. Baumgartner et al. utilized a biodegradable gelatin‐based foam and Zn electrodes to fabricate a capacitive pressure sensor for soft robotics and e‐skins^[^ ^8^ ^]^ (Figure 5d). However, engineered deformable structures such as pyramids render tunable pressure sensor characteristics for various material compositions.^[^ ^115^ ^]^ Boutry and co‐workers used this concept to render completely biodegradable (and bioresorbable) pressure sensors. In their first work, they fabricated a highly sensitive pressure sensors with arrays of Fe–Mg electrodes and a micropyramid patterned PGS dielectric.^[^ ^116^ ^]^ The sensor, embedded in PHAs, uses a pattern of 5 × 5 µm large pyramids (with 5 µm spacing) to achieve a sensitivity of 0.76 kPa^−1^ (relative capacity change) for pressures up to 2 kPa and 0.11 kPa^−1^ for pressures in the range of 2–10 kPa. Using a 5 × 5 electrode array, the sensors resolved the position of a fly, yet with large crosstalk between the sensors. Introducing stretchable POMaC as encapsulation for a similar pressure sensor, Boutry et al. fabricated a semi‐stretchable sensor system that measures pressure and strain independently.^[^ ^44^ ^]^ They use two patterned bottom electrodes (100 nm Mg on 50 µm PLLA films), which are relatively displaced upon deformation and measure tensile strain up to 15% (Figure 5b). The relative capacity change upon pressure is measured simultaneously between the top electrode and the closest bottom electrode (these electrodes have a fixed position under deformation). Distinguishing between tangential and longitudinal forces will also be necessary when replicating e‐skins for robotics. Hierarchical 3D structures of capacitive or resistive sensors is a promising solution to achieve robotic skins that mimic the function of human skin^[^ ^117^ ^]^ and, with the use of biodegradable materials, could sustainably upgrade our robotic creations.

Besides pressure and strain, the information on temperature and humidity (biodegradable material properties often depend on those two quantities) needs to be acquired to obtain a full picture of a robot's environment. Temperature sensors are best realized with materials that exhibit a positive electrical resistance change (positive temperature coefficient, PTC) with increasing temperature. Biodegradable metals, typical PTC materials, offer linear resistance change over a broad temperature range and are a common choice for temperature sensors. Salvatore et al. fabricated a deformable temperature sensor with serpentine shaped Mg traces encapsulated in Ecoflex.^[^ ^118^ ^]^ The resistive sensor area consists of 10 µm thick Mg traces with a resistance of 29 kΩ at room temperature, 130 times higher than the broader meander shaped connections (≈250 µm broad) (Figure 5e). The reported temperature coefficient of 2.45 × 10^−3^ K^−1^ is slightly lower than literature values for Mg thin films and results in a sensitivity of 0.2% K^−1^. Natural wax composites can be alternatively used to pure metals, as they allow easier fabrication—coating techniques or 3D‐printing are sufficient,—are bio‐derived and biodegradable, and often hydrophobic. Won and co‐workers introduced a bioresorbable mixture of candelilla wax and W particles (up to a W ratio of 0.4 vol%) that can be utilized as either an electrical connection (high W content) or temperature sensor^[^ ^119^ ^]^ (Figure 5f). In contrast to metals, the resistance of wax composites changes with temperature as the wax expands in volume, which increases the tunnel barrier of the incorporated conductive particles and hence results in a nonlinear temperature dependence. The performance is therefore limited to the melting point of the used wax, but usually gives sufficient results up to 45 °C. Baumgartner et al. prepared a metal free wax composite by mixing carnauba wax with carbon particles.^[^ ^8^ ^]^ The investigated temperature sensors were fabricated on a gelatin‐based elastomer showing a relative resistance change of 50% between 10 °C and 40 °C. Interestingly, the sensor established constant, nearly linear performance after the third heating cycle (between 10 °C and 40 °C), indicating a favorable segmentation of carbon particles.

Sensing of humidity requires two electrodes, typically patterned as interdigitated electrodes, and a substrate that responds (mechanically or electrically) to a humidity change. A frugal material choice is the use of paper as substrate, as the hydrophilic cellulose fibers absorb water in constant equilibrium with their environment. The ionic conductivity (and hence the impedance) of paper depends on the absorbed water and can be measured with two electrodes. Using this concept, Güder et al. built a humidity sensor from printed graphite ink on paper to monitor the breathing frequency via aspiration through a face mask.^[^ ^120^ ^]^ The substrate however can readily be replaced by any kind of material that exchanges water with its environment in equilibrium. Biodegradable hydrogels for example were utilized as humidity responsive substrate in combination with Zn electrodes, showing a relative impedance change of two orders of magnitude between 10% and 80% relative humidity^[^ ^8^ ^]^ (Figure 5g).

### 4.2 Output

Interaction between humans, animals, or robots is based on communication. Untethered forms of robots additionally need to communicate wirelessly, with humans over speech or visuals, or with other machines via data transmission. This eventually requires speakers, displays, and antennas such as for radio frequency (RF) coils, WiFi, or Bluetooth. Soft and stretchable forms of such components are highly challenging and currently drive major progresses for wearable electronics or biomedical applications.^[^ ^121^ ^]^ Biodegradable communication electronics, even nonstretchable ones, were yet only demonstrated with reduced complexity for electronic implants. However, extending these concepts for autonomous soft robotics will be sufficient for many applications that require to be sustainable but where recycling cannot be enabled.

**Figure 6:**
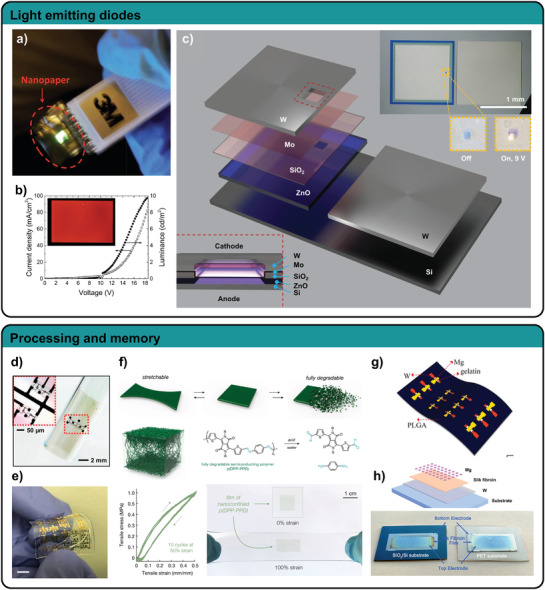
Bioresorbable and degradable solutions for LEDs, transistors, and memory devices. a) An OLED fabricated on nanocellulose paper. Reproduced with permission.^[^ ^122^ ^]^ Copyright 2013, Royal Society of Chemistry. b) Luminance–current–voltage characteristic of a solution‐processed bio‐OLED with a vitamin‐derived riboflavin tetrabutyrate emission layer. Reproduced with permission.^[^ ^124^ ^]^ Copyright 2017, American Chemical Society. c) Bioresorbable LED utilizing ZnO as emission layer. Reproduced with permission.^[^ ^125^ ^]^ Copyright 2019, Wiley. d) Foundry‐based CMOS logic based on bioresorbable Si transistors. Reproduced with permission.^[^ ^127^ ^]^ Copyright 2020, National Academy of Sciences. e) Organic transistors for biodegradable logics. Reproduced with permission.^[^ ^128^ ^]^ Copyright 2017, National Academy of Sciences. f) Stretchable and biodegradable transistors based on a physically confined p(DPP‐PPD) semiconductor. Reproduced with permission.^[^ ^130^ ^]^ Copyright 2019, American Chemical Society. g) Fully degradable resistive memory device using gelatin as switching layer. Reproduced with permission.^[^ ^132^ ^]^ Copyright 2020, IOP Publishing Ltd. h) Bioresorbable memory device based on silk fibroin as resistive layer and W and Mg as electrodes. Reproduced with permission.^[^ ^131^ ^]^ Copyright 2018, American Chemical Society.

In general, thin film electronics has four integration levels of sustainability. First, the dominant component, the substrate, is replaced with ecofriendly materials. Second, alternatives for the active materials, electrodes, or encapsulations are developed. Third, the whole device is rendered biodegradable or bio‐derived, with—the fourth level—sustainable processing tools and methods. LEDs fabricated on nanocellulose paper are an example of level one integration. Nanocellulose has favorable properties as it is highly transparent, mechanically robust but with a lower density as plastics, and offers a low thermal expansion coefficient.^[^ ^122^ ^]^ While Zhu et al. fabricated organic light emitting diodes (OLEDs) directly on the nanocellulose paper (**Figure** **6**a), Gomez and Steckl conducted the device fabrication on a silicon wafer before transferring it onto nanocellulose.^[^ ^123^ ^]^ Both approaches however used conventional materials for the actual OLEDs. Addressing this issue, Jürgensen et al. replaced the emission layer with vitamin‐derived riboflavin tetrabutyrate to introduce a bio‐derived active material (integration level two).^[^ ^124^ ^]^ The resulting OLED showed a maximum luminance of ≈10 cd m^−2^ with an emission peak at 640 nm (Figure 6b). The first completely transient and bioresorbable (inorganic) LED was recently published by Lu and co‐workers.^[^ ^125^ ^]^ Their device consists of oxygen vacancy doped n‐type ZnO(001) as emissive layer deposited on 12 µm thin n‐type Si(111) serving as substrate and anode (Figure 6c). An 8 nm thin layer of Mo serves as transparent cathode and thick W leads allow delivery of high currents. The diode emits light with a maximum optical power density of 0.7 mW cm^−2^ at 9 V and an intensity peak at 420 nm. Broad visible emission is observed likely due to defect states and allows red, green and blue emission with respective optical filters.

### 4.3 Switching: Transistors and Memory

The use of transistor technology to address large amounts of sensors or LEDs, or for the use as logics, memories, or active sensors is elementary for robots that require a higher degree of complexity. While inorganic transistors achieve high performance and can typically be integrated into small chips, organic transistors are potentially less expensive, can be printed on large scale, and—very promising for soft robotics—allow for intrinsically stretchable solutions.^[^ ^126^ ^]^ Bioresorbable or “green” transistor solutions are under investigation for more than a decade and reviewed in detail recently.^[^ ^3^, ^12^ ^]^ Similar to LEDs, previous research efforts were directed towards fabrication, compatible biodegradable substrates, dielectrics, or semiconductors (both inorganic and organic). More recently (2017), complete solutions progress towards fully degradable yet high performance complementary metal–oxide–semiconductor (CMOS) or pseudo‐CMOS circuits.

Chang et al. introduced materials and processing approaches that enable foundry‐processable water‐soluble CMOS circuits, including metal‐oxide‐semiconductor field effect transistor (MOSFET) inverters and logic ports^[^ ^127^ ^]^ (Figure 6d). In short, the transistors were fabricated with standardized foundry‐based processes and materials by an external company and then transfer printed onto biodegradable PLGA substrate. The transfer process starts with anisotropic (undercut) etching of Si to remove the chips from the wafer, followed by an individualized pick‐and‐place transfer with a microstructured polydimethylsiloxane (PDMS) stamp. Tungsten electrical connections were patterned as needed to achieve various logic circuits and encapsulated with another layer of PLGA. The transfer‐printed nMOSFETs have a typical performance (on/off ratio of ≈10^8^, threshold voltage 1.2 V, subthreshold slope of ≈100 mV decade^−1^, on‐resistance of ≈18 kΩ at gate voltage 5 V, carrier mobility 630 cm^2^ V^−1^ s^−1^) and dissolved within 10 d in an accelerated degradation experiment (96 °C in PBS). Degradation in more realistic environments can be assumed to take years, however the introduced methods show how the transition process toward sustainable electronics can be achieved with standard industrial processes.

Organic material approaches potentially offer faster degradation rates and provide performances that are sufficient for many applications. Lei et al. demonstrated pseudo‐CMOS inverters and logic ports utilizing synthetic poly(diketopyrrolopyrrole)‐phenylenediamine (PDPP‐PD) as organic p‐type semiconductor, aluminum (Al) oxide as dielectric, and Fe as electrical contact^[^ ^128^ ^]^ (Figure 6e). The transistors are fabricated on ultrathin (800 nm) nanocellulose paper, resulting in a total device thickness below 1 µm, which makes this approach interesting for ultra‐lightweight applications (millimeter sized robots) or whenever a high degree of flexibility is desirable. The transistor performance—although distinctly lower than inorganic transistors—is on par with nondegradable conjugated polymers (on/off ratio >10^4^, threshold voltage −5.75 V, carrier mobility 0.12 cm^2^ V^−1^ s^−1^). Yet, the devices completely dissolve within 30 d (room temperature in cellulase‐containing buffer at pH 4.6), dominated by the degradation of the organic semiconductor and the cellulose substrate. The authors note that longer stability could be achieved with suitable encapsulations.

Besides ultrathin or small electronics, intrinsically stretchable devices offer multifunctional integration (such building blocks contribute mechanical and electrical functionalities) into soft robotics. Chemically engineered semiconductors that are both stretchable and biodegradable are a rather new development with yet only few examples. Sugiyama et al. were upon the first to demonstrate stretchable, degradable, and semiconducting polymers.^[^ ^129^ ^]^ They synthesized a block copolymer consisting of stiff diketopyrrolopyrrole (DPP) units and insulating PCL units, rendering a semiconductor with >100% stretchability and a charge carrier mobility of 0.1 cm^2^ V^−1^ s^−1^. Surprisingly, the DPP content can be reduced to 10 wt% without influencing the field‐effect mobility. Tran and co‐workers achieved stretchability by embedding confined DPP nanofibrils in a PCL‐based bio‐PU.^[^ ^130^ ^]^ Their semiconducting polymer shows strain independent performance, being biaxial stretchable up to 50% strain (reversible) (Figure 6f). Balancing performance and DPP content resulted in charge carrier mobilities of 0.05 cm^2^ V^−1^ s^−1^ at 30 wt% DPP.

Switching between states and more importantly memory can also be built from resistive devices. Memristors have a simple structure (mostly metal‐insulator‐metal), low power consumption, and are low cost. While a number of transient memory devices were reported that dissolve in water—a recent review is given here,^[^ ^101^ ^]^—only a few of them are completely biodegradable, leaving nontoxic residues. Ji and co‐workers realized a biodegradable metal‐insulator‐metal device, using W and Mg as electrodes (80 nm each) and silk fibroin (120 nm) as switching layer^[^ ^131^ ^]^ (Figure 6h). The silk fibroin film was obtained from a silk cocoon and prepared in a three‐step process that includes boiling, dissolving, and spinning. The resistive switching mechanism of silk fibroin is still not fully understood, but the authors consider electrochemical activities (caused by the Mg electrode) to play a major part. Diffusion of Mg^2+^ ions may enhance the oxidation of silk fibroin, switching to a high conductivity oxidation state and the formation of conductive filament (SF‐Mg^+^ or SF^+^). A reset voltage reduces the oxidized filament and ruptures the conductive filament, setting the high resistive state. The resistive memory devices exhibit an on/off ratio >10^5^ and were addressed by switching between ±4 and 0 V, repeatedly for 100 times. The combination of thin layers of metal and silk fibroin result in fast device degradation within 24 h in PBS, however, this does not include the substrate. For practicability, the authors fabricated the resistive memory on nondegradable Si/SiO_2_ or PET substrates. Employing such devices in applications will further require biodegradable substrates (some examples are mentioned in this review) and suitable encapsulations to minimize influence of, e.g., humid environments.

Recently, Liu et al. fabricated a nonvolatile resistive memory on biodegradable PLGA as substrate (40 µm).^[^ ^132^ ^]^ Similar to the work of Ji et al., they used Mg (250 nm) and W (120 nm) as electrodes, but a thin (50 nm) gelatin film as switching layer (Figure 6g). The devices exhibit a reliable switching behavior with an on/off ratio of over 10^2^—even after 200 bending cycles— and long retention times of over 10^4^ s. Observing the degradation process in DI water, the device dissolved within 72 h, leaving only the PLGA substrate which usually degrades within weeks/months under physiological conditions. The use of biomaterials like silk or gelatin is an interesting approach for resistive memory devices but will require additional efforts towards material optimization (both mechanically and electrically), application‐oriented device designs, and integration with other electrical or robotic components.

### 4.4 Electronic Skins

Assemblies of multimodal sensor networks that mimic the functionality and feedback of natural skin are a research frontier in flexible and stretchable electronics. The integration of such e‐skins on the soft robotic body promotes machine–human interaction and autonomy through providing essential feedback from the robot's environment. Sustainable solutions for whole e‐skins are rare and mostly exist only for single sensors that require an external readout infrastructure. Recent developments, however, progress toward biodegradable sensor assemblies that can be worn on the skin with possible benefits for advanced healthcare, rehabilitation, and soft robots.

**Figure 7:**
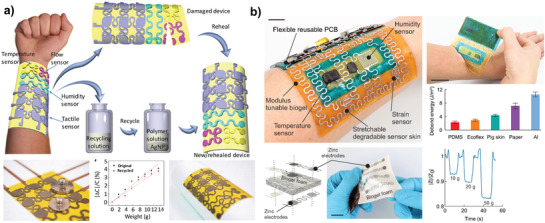
Recyclable and biodegradable e‐skins. a) Temperature, humidity, flow, and tactile sensors are fabricated on recyclable polyimine loaded with Ag nanoparticles. The e‐skin is bendable and differentiates touch input on the tactile sensor matrix. Reproduced with permission.^[^ ^133^ ^]^ Copyright 2018, American Association for the Advancement of Science. b) Soft and biodegradable e‐skin hosts various sensors that are autonomously operated by a multipurpose flexible PCB. After peel‐off of the self‐adhering biogel the PCB finds application on a new e‐skin, measuring pressure input at different locations. Reproduced with permission.^[^ ^8^ ^]^ Copyright 2020, Springer Nature.

Zou et al. developed a sustainable e‐skin concept based on recyclable (yet not biodegradable) materials and electronics.^[^ ^133^ ^]^ They developed a covalently bond polyimine thermoset that serves as a (bendable) substrate (Young's modulus, ≈1 GPa) and a polyimine/Ag nanoparticle composite as conductive/sensing material. Sensor structures were laser cut from conductive polyimine sheets and laminated on the nonconductive substrate. The e‐skin included resistive temperature, flow and humidity sensors (resistivity of conductive polyimine ranged from 0.02 to 0.12 Ω mm) and capacitive pressure sensors that were all addressed with external equipment (**Figure** **7**a). The conductive polyimine can both be healed or recycled through adding additional polyimine/Ag prepolymer and subsequent hot pressing, or dissolving it in its presolution and subsequent polymerization. Both methods can be performed at temperatures <80 °C and result in similar performance when comparing the original samples with the healed or recycled ones. The authors conclude that softer materials—necessary for on‐skin applications—might be achievable by changing the imine chemistry.

A soft, stretchable, and biodegradable e‐skin that can autonomously be operated on the body was recently demonstrated by Baumgartner and co‐workers.^[^ ^8^ ^]^ In their work they used a self‐adhering biogel (see Section 3.2) as biocompatible substrate to interface human skin and sensors based on 50 µm thin Zn foil and other resistive elements (Figure 7b). The sensors were addressed by a battery‐powered flexible PCB that hosts all read‐out electronics and (wireless) communication to send data to a smartphone. The authors envision the PCB to be recycled for multiple uses—a multipurpose architecture allowed application for two different sensor skin layouts,—while the sensor skins themselves are entirely biodegradable. The first e‐skin included temperature, humidity, and strain sensors that were connected with stretchable Zn meanders to measure temperature increase in the proximity of hot objects, aspiration, or deformation. A second e‐skin hosted a pressure sensor matrix to spatially resolve tactile feedback, using a capacitive sensor design with a deformable elastomer foam. Surprisingly, while the materials degrade in wastewater within a few weeks, the e‐skins were operational for over a year when stored under ambient conditions. Taking advantage of the thermoplastic properties of the gelatin‐based hydrogel, the authors introduced a rapid healing process that locally melts the gel and restores its functionality utilizing a laser engraving tool. Healed versus pristine films show no significant difference in their mechanical properties, while being healed within a few minutes under ambient conditions. This healing process enabled modular assembly of biogels with graded Young's modulus to establish substrates with strain isolated islands, or complex 3D shapes that are not easily achievable with common fabrication tools. Miniaturizing the biodegradable e‐skin concept for integration in autonomous soft robots and combining electronics and actuators with sustainable energy solutions is among the next steps to be taken.

## 5 Energy Sources

Powering actuators and e‐skins is one of the remaining challenges toward untethered, fully autonomous operation of (soft and lightweight) robots. Providing sufficient power and energy mainly determines their functionality, possible tasks, and (useful) applicability. The transformation from hard to soft nature‐inspired machines shifts design paradigms also for energy supplies. While it is common in conventional robotics to think of motors, sensors, and batteries as individual parts, distributed energy systems that closely interact with actuators and sensors are in close analogy to biological systems and more efficient for autonomous robots.^[^ ^134^ ^]^ Rethinking the design process of nature‐inspired electromechanical systems as a whole, creates untapped possibilities in our material choices, toward lightweight and low‐cost solutions with environmentally benign and abundant materials. Harvesting and storing renewable energy with ecofriendly energy solutions is the last puzzle of sustainable robotics with enormous benefits for autonomous operation, even in remote areas apart from any power grid, deep‐sea, or space.

**Figure 8:**
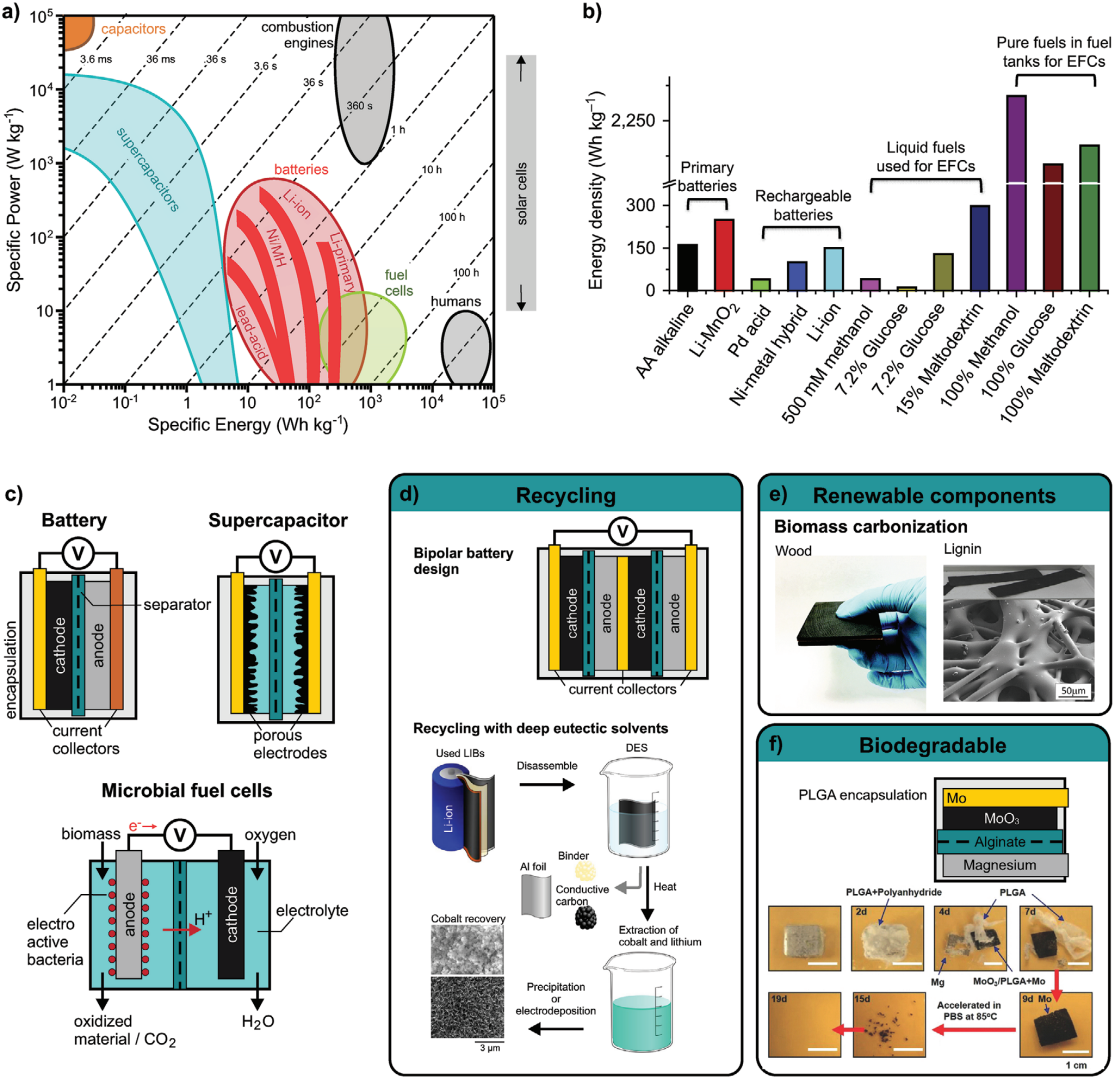
Sustainable energy supply. a) Ragone plot for different energy storage systems and comparison to combustion engines, humans and solar cells, which provide power up to 23 W g^−1^. Adapted with permission.^[^ ^135^ ^]^ Copyright 2018, Wiley. b) Energy densities of various energy materials and devices in comparison to enzymatic fuel cells. Adapted with permission.^[^ ^173^ ^]^ Copyright 2014, Springer Nature. c) Typical architectures of batteries, supercapacitors, and microbial/enzymatic fuel cells are multimaterial devices. d) Adapting these architectures to a bipolar battery design allows efficient recycling of, e.g., current collectors. Deep eutectic solvents provide an eco‐friendly recycling scheme for Li‐ion batteries. c,d) Adapted with permission.^[^ ^137^ ^]^ Copyright 2019, Springer Nature. e) Carbonized biomass finds application as renewable electrode material in supercapacitors or batteries. (Left image) Reproduced with permission.^[^ ^155^ ^]^ Copyright 2017, Royal Society of Chemistry. (Right image) Reproduced with permission.^[^ ^143^ ^]^ Copyright 2013, Wiley. f) Biodegradable battery design and degradation process. Reproduced with permission.^[^ ^146^ ^]^ Copyright 2018, Wiley.

How much energy a soft robot needs, depends on size, weight and application. Humans—in essence a role model for nature inspired machines—have an average weight of 62 kg and consume 8000 kJ per day, resulting in roughly 36 000 Wh kg^−1^ available specific energy. This high amount of specific energy is unmatched by typical (electrical) energy storage systems and stems from the efficient biochemical energy conversion found in our body. Artificial machines however, cover a large spectrum of specific power, which ranges over 10^5^ W kg^−1^. In comparison, our power output at rest is about 85 W, and even athletes can only deliver 400–600 W for extended periods, which results in a specific power density of 1–10 W kg^−1^. Yet, this amount of specific power is enough to accomplish a myriad of tasks, even though at a lower speed. A comparison of different classes of (electrical) energy storage systems can be visualized by a Ragone plot,^[^ ^135^ ^]^ where the specific power is drawn versus the specific energy of a system (**Figure** **8**a). The response time (the ratio of specific power and specific energy) is characteristic and divides electrical energy storage into capacitors, supercapacitors, batteries, and fuel cells. For the latter two, the specific energies achieved from typical representatives of that energy storage classes are depicted in Figure 8b. In this chapter, we discuss sustainable materials and design routes for these energy storage systems and extend the discussion to renewable energy harvesting via solar cells and biofuel cells.

### 5.1 Batteries

Batteries represent the gold‐standard energy storage for autonomous robots, portable devices and machines. A typical battery configuration comprises a cathode and an anode, separated by an ion‐conducting membrane, an electrolyte solution, current collectors, and an encapsulation/packaging (Figure 8c). Sustainable battery technology can focus on replacing individual battery components with renewable, environmentally benign or biodegradable materials, or on recycling critical components if ecofriendly solutions are not feasible. Successful solutions probably will utilize both approaches, recyclable and bio‐derived components, to achieve a practical compromise between sustainability and performance. Applications that do not allow access to the robotic body, such as when operated in remote areas or in vivo, require completely transient and biodegradable energy storage. In this section we discuss these options—from recycling, renewable materials, to biodegradable devices—for batteries (Figure 8d–f).

Recycling of battery components is only efficiently achievable if the materials or components are economically valuable—much more than environmentally valuable—and the recycling process comparatively cheap (see discussion in Section 2.2). The utilization of lithium (Li) batteries—in smartphones, portable computers, or electric vehicles—rapidly increased over the past two decades, yet their recycling poses an ongoing challenge (although Li battery recycling was already reviewed in detail in 2008^[^ ^136^ ^]^) and is far from being industrially employed.^[^ ^13^, ^18^ ^]^ This is mainly because each manufacturer develops different cathode compositions ranging from Li–nickel–cobalt–Al (Li–Ni–Co–Al) oxides to Li–Ni–Mn oxides or Li–Fe phosphates, which require high‐energy‐demanding extractive metallurgical processes for recycling individual metals. Current research addresses this issue by introducing new recycling strategies that are efficient, applicable to various cathode chemistries, and require less energy. Tran and co‐workers developed a recycling scheme for Li‐ion battery cathodes based on deep eutectic solvents (DES)^[^ ^137^ ^]^ (Figure 8d). DES—in their work they used a 1:2 molar mixture of choline chloride and ethylene glycol—are a class of ecofriendly compounds with a high capability of dissolving metal oxides. In their recycling scheme, they first disassembled a Li‐ion battery and dissolved the cathode in DES, obtaining a leachant with Li–Co oxide and, through filtering, battery components such as Al foil, binder, and conductive carbon. Co and Li metals were extracted from the leachant either through precipitation or electrodeposition, allowing reutilization of these valuable materials with leaching efficiencies of ≥90%. New battery technologies, such as sodium ion or solid‐state batteries, will shift the focus to different material strategies and, with a growing awareness for sustainability, employ green battery chemistries or recycling strategies right from the beginning. Liu et al. reported a cell design that allows easier recycling of sodium ion battery electrodes.^[^ ^138^ ^]^ The design utilizes a bipolar electrode structure of shared Al foil and Na_3_V_2_(PO_4_)_3_ electrodes that are recycled with an efficiency of ≈99.1% and 100%, respectively without the release of toxic wastes (Figure 8d). Cathode materials were reprocessed, closing the fabrication loop for sodium ion batteries.

Replacement or recycling of batteries integrated in soft robotic bodies could pose an additional challenge. This is mainly because autonomous soft robotic systems can achieve a much higher efficiency when the energy storage—much like as for any other living being—is distributed over the whole body and, when considered in soft form factors, batteries simultaneously comprise electrical and mechanical functionalities. If the whole robotic body is a battery, each type of robot will require unique battery shapes and systems that might be difficult to recycle on a large scale. In this case, the use of abundant renewable materials and components is a solution to progress sustainable battery technology. Wood‐based materials, such as cellulose and lignin, are extensively researched as renewable materials for energy storage systems and reviewed in detail in the past years.^[^ ^139^, ^140^, ^141^ ^]^ For Li‐ion batteries, bioderived components are mainly integrated as separator, electrode (when carbonized) or binder material, but were also included into the cathode^[^ ^142^ ^]^ and anode^[^ ^143^ ^]^ (Figure 8e, right). Delaporte et al. recently reported on a flexible Li‐ion battery that utilizes cellulose fibers as binder and support for the electrode and active materials when mixed with different carbons or redox active materials (such as LiFePO_4_ (LFP), Li_4_Ti_5_O_12_ (LTO), or organic 3,4,9,10‐perylenetetracarboxylic dianhydride (PTCDA)).^[^ ^144^ ^]^ Their best battery achieved a capacity of 160 mAh g^−1^ (without encapsulation) with LTO electrodes and about 80 mAh g^−1^ with PTCDA electrodes.

Completely biodegradable batteries further eliminate issues that arise from toxic‐degradation products, are often from low‐cost renewable resources and, when made from bioresorbable materials, can be implanted into the human body. The latter is also the main driving force behind activities towards transient power sources and was recently reviewed by Jia and co‐workers.^[^ ^126^ ^]^ While the performance of current biodegradable batteries is sufficient to drive low‐energy electronic implants for a suitable time scale, their performance metrics (energy density, capacity, rechargeability) are still far from being competitive to state‐of‐the‐art Li‐ion batteries. Indeed, biodegradable energy storage and wireless communication are two main bottlenecks to realize fully transient electronic devices, and likewise a major challenge for sustainable robotics. Furthermore, embodiments of soft and stretchable batteries are highly desired for autonomous soft robotics and electronics but—although nondegradable soft batteries are increasingly reported—do not exist as biodegradable. Despite these current limitations, biodegradable energy materials have a great potential as low‐cost green energy storage for all kinds of sustainable electrical devices.

A typical approach to render a biodegradable primary cell is to use two electrodes with biodegradable metals, such as Mg, Mo, Zn, or Fe, with different electrochemical potentials. Most promising are batteries composed of Mg and its alloys as Mg has fast degradation rates and high theoretical capacity of 2.2 Ah g^−1^. One of the first biodegradable batteries was reported 2014 by Yin and co‐workers, who used Mg as anode and Mo, W, or Fe as cathode.^[^ ^145^ ^]^ For a single cell with 50 µm thick Mg foil as anode and 8 µm thick Mo foil as cathode the battery delivered 142 mAh g^−1^ when discharged with 0.1 mA cm^−2^ (normalized to anode and cathode mass and area). Arranging a battery as a four‐compartment cell, using a Mo paste (Mo mixed with water‐soluble sodium carboxymethyl cellulose glue) as separator and polyanhydride as encapsulation, resulted in a stable voltage output of ≈1.5 V for up to 6 h. This battery degraded partially within 11 d (37 °C in PBS), leaving behind only the Mo foils, which degraded in accelerated conditions (85 °C in PBS) after additional 8 d. Replacing the Mo cathode with MoO_3_ resulted in a higher open circuit voltage of 1.6 V and higher specific power, yet with reduced specific energy^[^ ^146^ ^]^ (Figure 8f). Other biodegradable batteries based on Mg anodes achieve higher performance, but on the cost of biodegradability, as they often utilize nondegradable current collectors (such as Au, Cu, or polypyrrole). Therefore, finding a compromise between performance, recycling, and biodegradability is a challenge that follows application requirements. Here, rechargeable sodium‐ion batteries (SIBs) are a promising battery technology due to their high energy density, low cost, high (charging) cyclability, and safe (less flammable) constituents. Typical SIBs have the same architecture as Li‐ion batteries, but often consist of more abundant materials. Typically, nongraphitizable carbons (hard carbons) are utilized as anode, metal oxides containing abundant metals (Na, Fe, Mn, Mg) as cathode, both aqueous or non‐aqueous electrolytes can be used, and Al serves for the current collectors.^[^ ^147^ ^]^ While no completely biodegradable or renewable SIB was demonstrated so far, research efforts towards sustainable SIB technology may lead to an ecofriendly battery solution. Anodes are readily fabricated from renewable sources—as hard carbons can be obtained from biomass‐waste,^[^ ^17^ ^]^—or from biodegradable ones when synthesized from organic polydopamine,^[^ ^148^ ^]^ and Kim et al. reported on biodegradable melanin‐based cathodes for SIBs, yet with reduced energy density.^[^ ^149^ ^]^ Furthermore, ecofriendly electrolytes and biodegradable current collectors are required to achieve fully biodegradable SIBs.

### 5.2 Supercapacitors

Supercapacitors are a class of energy storage devices that bridge the gap (in terms of specific energy and power) between common dielectric or electrolytic capacitors and batteries. As they feature higher specific power (and lower energy density) compared to batteries, supercapacitors are useful to deliver short energy pulses for communication devices, or to stabilize the output voltage of a battery. Robots that use diverse energy storage systems instead of battery‐only solutions benefit from a power management that is adjusted to changing workloads, leading to a more efficient use of available energy.^[^ ^150^ ^]^ Two types of supercapacitors exist: 1) electrical double‐layer capacitors (EDLCs) and 2) pseudocapacitors. The first accumulate charges at the double layer building up at electrode–electrolyte interface. The second feature rapid and reversible faradic redox reactions that occur at the surface of the electrodes. In both cases, the supercapacitor architecture is simpler than those of batteries, commonly consisting of electrodes, electrolyte, and a separator. The simple architecture and the absence of necessary chemical reactions for EDLCs, eases the implementation of biodegradable solutions and materials.

Carbonized biomass is intensively investigated as ecofriendly electrode material for EDLCs due to its porous structure, electric conductivity, abundance of precursor materials, and chemical inertness (recently reviewed^[^ ^151^, ^152^ ^]^). While precursor materials range from fruits, plants, to microorganisms, the supercapacitor performance is mainly influenced by the resulting specific surface area, surface morphology, and graphitization degree. Ma and co‐workers prepared nitrogen‐doped porous carbon from potato waste residues.^[^ ^153^ ^]^ The surface morphology and nitrogen content were controlled by the carbonization temperature, resulting in capacitance values of 255 F g^−1^ at 0.5 A g^−1^ (normalized to electrode mass). The supercapacitor achieved a good capacitance retention of 93.7% after 5000 cycles in 2 m potassium hydroxide (KOH) electrolyte. To increase performance, Lu et al. developed a biomass treatment method that is applied before carbonization to enhance porosity, conductivity, and specific surface area.^[^ ^154^ ^]^ In their approach, they “drilled” holes into lotus plant biomass with H_2_O_2_, oxidizing cellulose and hemicellulose to produce rich mesopores. Carbonizing the structures at high temperatures leads to a high activated carbon ratio and results in a specific capacitance of 340 F g^−1^ at 0.5 A g^−1^ and high specific energy densities of 23.33 to 16.67 W h kg^−1^ (normalized to electrode mass). The electrodes in 3 m KOH electrolyte achieved 98% capacitance retention after 10 000 cycles. We note that the energy density of standalone devices for both approaches would be considerably lower, if the electrolyte, encapsulation, and separator (necessary to increase the operating voltage window and prevent electrolysis) were included in the weight calculation. A complete wood‐based supercapacitor was demonstrated by Chen and co‐workers^[^ ^155^ ^]^ (Figure 8e, left). The assembled devices from carbonized wood sheets (the cathode was additionally electrodeposited with MnO_2_) using a thin wood‐based membrane as separator and a gel electrolyte containing 1 m Na_2_SO_4_. While they do not report gravimetric values for energy density and capacitance, they achieved a capacitance retention of 93 % after 10 000 charge–discharge cycles.

The simple structure allows also for soft and stretchable forms of supercapacitors with promising potential for medical applications—as recently reviewed^[^ ^156^ ^]^—or soft robotics. Lee et al. reported on fabrication strategies and materials for biodegradable and stretchable (pseudo‐capacitive) supercapacitors. Their design employed a serpentine shaped Mo wire, in PVA‐based electrolytes and POC as elastic encasing. To enhance the capacitance, they anodized the pure Mo wire in electrolyte to form a thin, slightly porous layer of MoO_2_, resulting in ≈170 mF g^−1^ at a current density of 2 mA g^−1^ (normalized to the mass of the whole device) and a capacitance retention of 97% after 5000 charge–discharge cycles. The supercapacitor showed stable performance under bending and stretching up to 50% strain and remained functional when immersed in DI water for up to 12 d. Even though the performance characteristics are low compared to rigid biodegradable supercapacitors, arrangements that use multiple wires, coils, or sponges and less encapsulating material would greatly boost specific capacitance and energy density. Wu et al., for example, demonstrated a sponge‐like electrode from carbonized watermelons that were loaded with Fe_3_O_4_ nanoparticles to render magnetite carbon aerogels.^[^ ^157^ ^]^ This electrode achieved a high capacitance of 333.1 F g^−1^ at a current density of 1 A g^−1^ (normalized to the electrode weight) while being soft and reversibly compressible to 50% strain.

### 5.3 Solar Cells

Besides batteries and capacitors as energy storage devices, mobile applications require continuous flow of energy and recharging. When a connection to the power grid is not available, energy harvesting from renewable abundant sources is the key for a sustainable energy supply. Solar cells in particular represent the technologically most advanced energy harvesting system for mobile appliances. Robots equipped with photoactive materials can either directly be actuated with light^[^ ^158^, ^159^ ^]^ or recharge their storage for later use. However, utilizing solar power for sustainable autonomous robotics is challenging. First, while the energy source is green, the used solvents and designs can contain components that are harmful for the environment and therefore require rigorous waste management. Second, of‐the‐shelf solar panels are too heavy, with too low power per weight metrics to allow efficient use for autonomous robotics.

**Figure 9:**
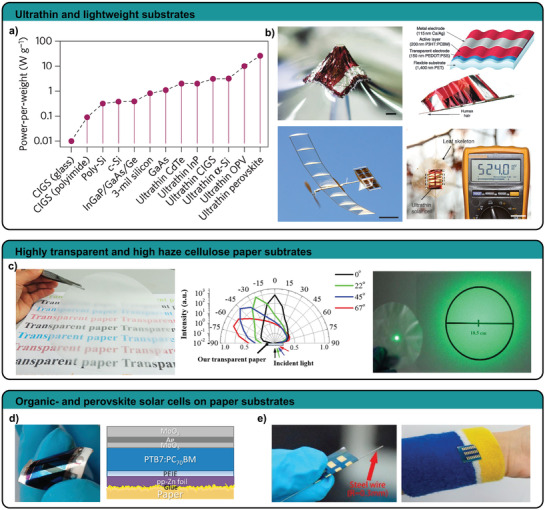
Sustainable concepts for solar cells. a,b) Organic‐ and perovskite solar cells fabricated on ultrathin (≈1 µm) plastic substrates save material usage and increase power per weight metric, which allows autonomous operation of a model plain. a) and b) (lower left and lower right) Reproduced with permission.^[^ ^14^ ^]^ Copyright 2012, Springer Nature. b) (upper left and upper right) Reproduced with permission.^[^ ^160^ ^]^ Copyright 2015, Springer Nature. c) Transparent nanocellulose paper increases solar cell efficiency by providing a high optical haze. Reproduced with permission.^[^ ^164^ ^]^ Copyright 2014, American Chemical Society. d) Ecofriendly solar cells based on organic materials and e) perovskites fabricated on flexible paper substrates. d) Reproduced with permission.^[^ ^166^ ^]^ Copyright 2014, American Chemical Society. e) Reproduced with permission.^[^ ^168^ ^]^ Copyright 2019, Elsevier.

A strategy that addresses high power per weight solar cells involves ultrathin substrates. Kaltenbrunner et al. fabricated organic and perovskite solar cells onto 1 µm thick PET foil as substrate, reducing the overall weight and waste by two orders of magnitude (at least waste that stems from substrate and encapsulation).^[^ ^14^, ^160^ ^]^ The perovskite solar cell yielded a record high power density of 23 W g^−1^ (**Figure** **9**a), twice what is commonly achieved by jet engines, which was sufficient to power a model airplane with eight solar panels (roughly 2 × 2 cm each) (Figure 9b). Such extreme lightweight power sources are also of interest for soft and lightweight robotics, ranging from millimeter sized crawlers to any larger design that provides enough surface area. Combination with energy storage systems, such as supercapacitors, allows energy supply from skin‐like devices whenever sunlight is not available.^[^ ^161^ ^]^

Beyond reducing the substrate thickness to reduce waste and materials costs, plastic substrates can be replaced with biodegradable materials from renewable sources. Paper or nanocellulose are possible green substrate solutions that allow for large scale, low‐cost fabrication via printing.^[^ ^162^, ^163^ ^]^ Nanocellulose paper has some favorable attributes for solar cells, as those papers combine high optical transparency and high optical haze, which increases light scattering and absorption into the active materials (Figure 9c). Fang and co‐workers fabricated a wood‐based nanocellulose paper with a transparency of 96% and optical haze of 60%.^[^ ^164^ ^]^ They utilized a 2,2,6,6‐tetramethylpiperidine‐1‐oxyl/sodium‐bromide/sodium‐hypochlorite (TEMPO/NaBr/NaClO) oxidation system to introduce carboxyl groups into the cellulose, which weakens the hydrogen bonds of cellulose fibrils. This treatment results in swelling and collapsing of the wood fibers to a paper with high packing density. Additionally, considering the boost of optical performance the authors report better mechanical properties compared to normal paper with an increase of the ultimate tensile strength from 8 to 105 MPa.

The use of biodegradable, cellulose‐based substrates in combination with solution processable active materials can greatly reduce material costs and environmental impact. However, the achieved efficiencies are considerably low when compared to Si‐based solar cells that reach efficiencies as high as 44%. For the first solar cell that was printed on paper Hübler et al. reported an efficiency of 1.3% in 2011.^[^ ^165^ ^]^ The challenges here are complex and do not only address the solar cell substrate but also the efficiency and sustainability of the active layer, the transparent top‐electrodes, and the overall stability with respect to air, humidity and temperature. In 2014, Leonat et al. reported on an indium‐tin‐oxide (ITO) free organic photovoltaic cell (OPV) on paper with an efficiency above 4%^[^ ^166^ ^]^ (Figure 9d). They used cold‐lamination of a thin polypropylene‐Zn layer on printing paper to produce a nonporous conductive electrode with high conductivity and standard OPV materials. The semitransparent top electrode, composed of a MoO_3_/Ag/MoO_3_ trilayer, served as replacement for the more common ITO. More recently (2019), Rawat and co‐workers optimized the fabrication process for OPVs on paper, utilizing a polyvinyl formal layer to smoothen glossy paper and polystyrene sulfonate doped poly(3,4‐ethylenedioxythiophene) (PEDOT:PSS) as transparent electrode.^[^ ^167^ ^]^ With their design they reached an efficiency of ≈6%. Besides organic materials, Li et al. fabricated a perovskite solar cell on 25 µm thick cellophane demonstrating an efficiency of ≈12%^[^ ^168^ ^]^ (Figure 9e). They deposited a TiO_2_/Ag/TiO trilayer as a semitransparent electrode on cellophane, which resulted in good bending stability, even after 1000 repeated bending cycles.

Additionally, to select more ecofriendly materials for solar cells, the fabrication process itself typically involves toxic chemicals and solvents. Research activities towards a “green” chemistry for solar cells or more generally sustainable fabrication schemes for semiconducting materials represent a field of their own.^[^ ^169^, ^170^ ^]^ Using low‐toxic solvents for OPVs does not necessarily result in compromises in performance. Hong and co‐workers demonstrated that with a small modification of the side‐chain of nonfullerene acceptors, OPVs can be fabricated with o‐xylene or tetrahydrofuran as processing solvents while achieving an efficiency of over 15%.^[^ ^171^ ^]^ Their approach is compatible with blade coating fabrication, promoting OPV mass production with more environmentally benign solvents. Still, sustainable solar cell fabrication requires more efforts toward optimization of ecofriendly materials and processing solutions, biodegradable substrates with improved shape stability in realistic environments, and—for robotics—high power per weight metrics and flexible or stretchable form factors.

### 5.4 Microbial Fuel Cells

Beyond typical renewable energy sources such as sunlight, wind, thermal energy, and vibrations, the generation of electricity from waste through biological organisms is an unconventional, yet useful energy harvesting solution. Microbial fuel cells (MFCs, or enzymatic fuel cells EFCs) utilize microbially catalyzed anodic, and microbial/enzymatic/abiotic cathodic electrochemical reactions to directly harvest energy from abundant sources such as wastewater,^[^ ^172^ ^]^ sugars,^[^ ^173^, ^174^ ^]^ or methane.^[^ ^175^, ^176^ ^]^ Indeed, MFCs represent the only existing technology that can do so, without the need of additional energy. The broad range of resources is based on the impressive diversity of electroactive microorganisms and on the conditions in which they function.^[^ ^177^ ^]^ For autonomous self‐sustainable robotics, these concepts are promising, as they open up new untapped energy sources that are richly available in remote areas. MFCs comprise also a higher energy density than batteries and more closely resemble the energy storage mechanisms in the human body. Yet, they deliver low specific power, which hampers their employment to drive strong electric motors but possibly unlock their full potential in combination with soft or lightweight robotics.

Even when robotic demonstrations powered by MFCs are rare, autonomous lightweight robots were demonstrated. Eco‐Bot II is a simple lightweight robot on wheels that progresses with the power of eight MFCs in series.^[^ ^174^ ^]^ It is equipped with photodiodes to follow a programmed pathway and can monitor and transmit temperature data via radio frequency. While the MFCs harvest energy from onboard sugar‐based solutions, the greater potential for MFCs lies within untapping resources available in the robot's environment. Philamore et al. envision a robot technology that works, eats and proceeds, similar to us naturally occurring creatures.^[^ ^178^ ^]^ They demonstrated a swimming robot that opens its “mouth” to activate the energy harvesting process and start propagation. To do so, the MFCs harvested energy from sewage water and charged several supercapacitors which drove a DC‐motor. Alternatively, they employed soft robotic actuators to compare energy conversion efficiencies—an ionic polymer metal composite (IPMC) and a shape memory alloy (SMA) actuator, as these actuators are biocompatible and have low operating voltages (1–3 V). The conversion efficiency (ratio of mechanical energy delivered by the actuator and electric energy produced by the MFC) was around 2.8–3.8 % for the DC‐motor and one magnitude lower (≈0.2%) for the soft robotic actuators. Boosting the efficiency to progress energy autonomous soft robots will require rigorous efforts in optimization of the whole energy supply/actuation system.

## 6 Special Applications

So far, plenty of material approaches can contribute to a more sustainable future of robotics. They range from recycling of valuable components, utilization of renewable base‐materials, and totally degradable solutions, often showing benefits and drawbacks with respect to ecofriendliness, performance, and costs. Material optimization is strongly application driven—biodegradable approaches mainly serve in vivo applications, recycling is utilized for energy storage, or renewable materials replace single use commodity plastics,—which involves additional challenges for soft robotics. For robots the material choices, robot designs, and performance parameters need to be closely adapted and optimized for specific tasks, environments, and economical concerns,—in other words—similar tasks performed in different environments might require completely different material choices. Autonomous robots—very much alike humans—that are highly adaptive, operate in various environments and are composed of ecofriendly materials are still illusive, yet the future of the field. This final chapter highlights designs of soft or lightweight robots or robotic materials, from low‐cost approaches to in vivo actuation.

### 6.1 Paper‐Based Robots

**Figure 10:**
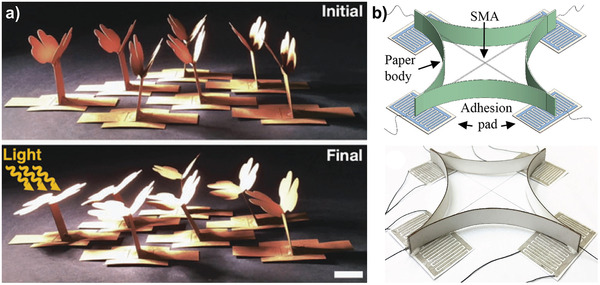
Paper‐based robots. a) Plant‐shaped paper structures use light responsive hydrogels to adjust their position toward higher light intensities. Reproduced with permission.^[^ ^179^ ^]^ Copyright 2020, Mary Ann Liebert Inc. b) Climbing robots using electroadhesion and SMAs to propagate on walls. Reproduced with permission.^[^ ^181^ ^]^ Copyright 2018, IEEE.

A frugal approach for extremely low‐cost, rapidly prototyped robotics is the use of paper. Cutting and folding—Kirigami and Origami are impressively powerful tools that repeatedly find application across many scientific fields—are fast processing steps that shape paper into functional structures, including stretchable ones. Cezan et al. combined laser‐cut paper with light responsive hydrogels as actuators to build self‐regulating plant robots.^[^ ^179^ ^]^ Inspired by the natural heliotropism of plants, these plant‐shaped robots change their orientation towards a light source and imitate folding and unfolding of flowers (**Figure** **10**a). The authors tuned the swelling/deswelling kinetics of hydrogels to respond to a single light stimulus; in their implementation illumination results in an increased shrinkage. Installing these stimuli responsive hydrogels at paper folds causes the paper to bend with respect to the adjusted light intensity. Such a strategy might be useful—as the authors demonstrate—to optimize the orientation of a solar panel adjusted onto such a plant‐robot to increase power output, without needing any additional energy input than the light source. Optimizing the swelling kinetics by using thin anisotropic structures allows film based robots with various locomotion patterns.^[^ ^180^ ^]^ In another demonstration of a paper‐based robot, Wu and co‐workers designed an electroadhesion climbing robot.^[^ ^181^ ^]^ For their robot, they utilized four printed electrodes on paper that are kept in position with a flexible paper frame. Two SMA artificial muscles are attached crosswise to the frame to manage its propagation (Figure 10b). The control electrodes, including voltage amplifiers and battery, are connected with sufficiently long wires to let the robot climb without immediate restrictions but are not meant to be carried themselves. This semi‐tethered robot is able to climb in two directions with an average speed of 1 mm s^−1^.

### 6.2 Biodegradable Pneumatic Actuators

Soft pneumatic actuators are successfully established throughout the soft robotics community due to (relatively) large actuation forces and strains, fast response time, and simple design. In essence, such actuators require a soft body (e.g., tube‐like or origami‐based designs), some sort of engineered restrictions to program the robot's locomotion, and a source of pressurized air or vacuum. While the first soft pneumatic actuator was already invented in 1957 by Joseph Law McKibben—also known for his contribution to the development of the atomic bomb,—the field of soft robotics started to get increasing attention around the 2010s. Yet, ecofriendly material approaches were addressed only recently (2017), using biodegradable elastomers (see Section 3.2) for pneumatic actuators.

**Figure 11:**
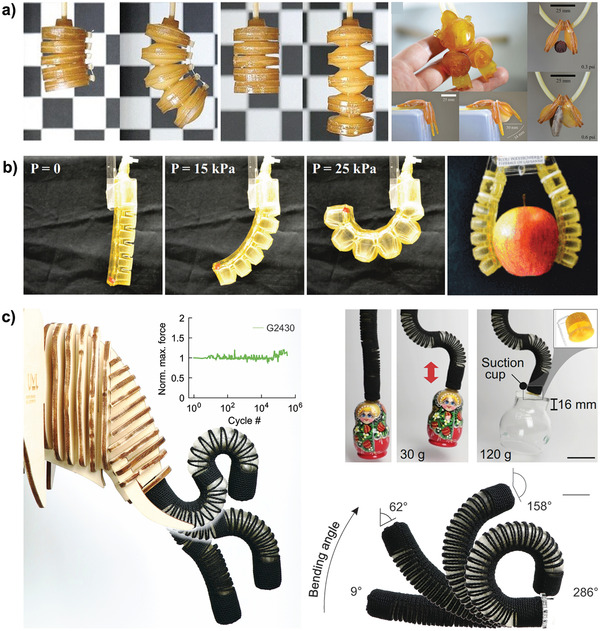
Biodegradable pneumatic actuators. a) A PGS‐based pneumatic actuator with bending and extension mode serves as a basis for frog‐shaped robots and grippers. Reproduced with permission.^[^ ^38^ ^]^ Copyright 2017, Springer Nature. b) Edible actuator and gripper fabricated from a gelatin‐glycerol hydrogel. Reproduced with permission.^[^ ^58^ ^]^ Copyright 2017, IEEE. c) Gelatin‐based actuator with a cotton‐fiber reinforcement to establish s‐shaped and u‐shaped movements with a repeatability of over 330 000 actuation cycles. A suction cup on the tip allows for grasping diverse objects (actuator weight, 35 g). Reproduced with permission.^[^ ^8^ ^]^ Copyright 2020, Springer Nature.

Walker and co‐workers utilized PGS‐CaCO_3_ elastomer sheets (157% to 242 % ultimate strain) for various pneumatic actuators, grippers, and simple robotic prototypes.^[^ ^38^ ^]^ They laser‐cut PGS disks from the sheets, which they laminated together using cyanoacrylate glue. Additional structuring of the elastomer surface gave control over the cyanoacrylate spreading and allowed fabrication of inflatable connected chambers. Two actuator designs, assembled from 3 to 5 chambers, were proposed—one for uniaxial extension and the second for bending motion (**Figure** **11**a). The uniaxial design achieved an extension of 35–50% strain with an applied pressure of 5–7 kPa and a blocking force of over 1 N in the same pressure range (0.45 N for the bending actuator). Grippers were manufactured from separately inflatable chambers to hold delicate objects like a raspberry. While the authors do not report maximum actuation cycles for a single actuator, the material itself withstands over 100 repeated stretch‐release cycles with only little fatigue, being a promising solution for applications with intermediate operation time. Noteworthy, the calculated CO_2_ emission during the synthesis and fabrication process of 1000 small PGS‐CaCO_3_ actuators equals the emission of a small car driving one mile.

Based on a gelatin‐glycerol hydrogel (150% ultimate strain), Shintake et al. demonstrated a fully edible monolithic bending actuator^[^ ^58^ ^]^ (Figure 11b). The actuator was cast in a two‐step molding process, first molding inflatable structures and second closing these structures with a thicker gelatin‐glycerol sheet to achieve actuator bending. Applying a 25 kPa pressure, the 90 mm long actuator achieved a bending angle of 170° and a (for this type of actuator typical) blocking force of 0.3 N. With their actuator, the authors were able to demonstrate 13 successive actuation cycles, mainly limited by its moderate stretchability and thin inflating structures. A gripper, designed from two opposing actuators, was utilized to grab and move different fruits, from apples to softer tangerines, and rigid objects of various shapes. The easy access and great processability of gelatin‐based gels inspired a student's group from the Haverford school (USA) to fabricate similar pneumatic actuators from commercially available confectionary gels.^[^ ^182^ ^]^ Despite efforts toward improving such gels, the fabricated actuators still have a limited lifetime of about 10 actuation cycles. Yet, this project demonstrates how sustainable technology can readily be integrated into education, focusing on scientific approaches that include sustainability as a key metric from early stages on.

Greatly improving the performance of biodegradable pneumatic actuators, Baumgartner et al. developed a gelatin‐based biogel that endures larger ultimate strain (>300%), is resilient against dehydration and easy to process.^[^ ^8^ ^]^ They fabricated a tube‐shaped actuator that uses a crocheted fiber reinforcement (cotton fibers) to adjust the motion patterns and designed actuators with u‐shaped and s‐shaped bending (Figure 11c). The u‐shaped bending actuator achieved 286° bending angle at 50 kPa applied pressure (limited by self‐collision) and 14.7 N blocking force at 102 kPa when the movement is restricted to the tip. Compared to existing biodegradable actuators the authors were able to increase the number of maximum actuation cycles by orders of magnitude to over 330 000 repeated cycles, progressing ecofriendly technology toward industrially relevant applications. Although being biodegradable in aqueous environments, this type of actuator performed near to 2000 cycles underwater utilizing suitable hydrophobic coatings. The s‐shaped bending actuator in combination with a suction cup could lift a 120 g heavy object for a distance of 16 mm (actuator weight 35 g) and grab various objects from soft to hard. Additionally, the authors attached a biodegradable pressure sensor onto the actuator tip to measure collision with other objects and therefore increase its applicability. While the authors envision biodegradable grippers to harvest produce or use edible robotics for prey imitation, further improvements are required—such as elastic hydrophobic coatings to increase outdoor lifetime or more complex integration of electronic control circuits—to increase the actuators versatility.

### 6.3 Milli‐ and Microrobots for In Vivo Applications

**Figure 12:**
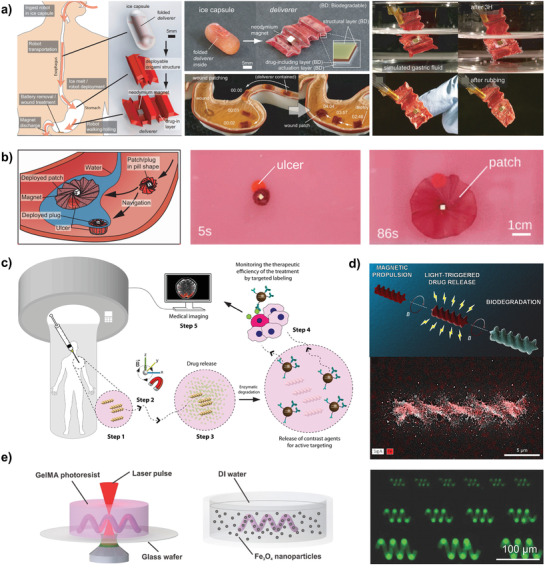
Bioresorbable milli‐ and microbots. a) Gastral robot that is delivered to the stomach when packaged in an ice capsule. The robot unfolds in the stomach where it is operated through an external magnetic field and degrades when its task is achieved. Reproduced with permission.^[^ ^183^ ^]^ Copyright 2016, IEEE. b) Wound patching robot is (magnetically) delivered to an ulcer in the stomach where it unfolds through an engineered swelling mechanism. Reproduced with permission.^[^ ^9^ ^]^ Copyright 2018, IEEE. c) Microbots are designed as drug carriers which propel upon application of an external magnetic field. Reproduced with permission.^[^ ^185^ ^]^ Copyright 2019, American Chemical Society. d) Drug carrier microbots usually consist of a biodegradable polymer matrix loaded with Fe nanoparticles. The degradation process and release of drugs can be initiated by light illumination. Reproduced with permission.^[^ ^186^ ^]^ Copyright 2018, American Chemical Society. e) Two photon lithography is used to 3D‐print microbots in a micrometer scale, followed by soaking them in a Fe nanoparticle dispersion. Reproduced with permission.^[^ ^187^ ^]^ Copyright 2018, Wiley.

Remotely controlled miniature robots that operate in vivo to carry out or assist clinical procedures are a long‐term goal in the field of robotics. Bioresorbable materials are here favorable due to their natural disintegration inside the body, eliminating the need of a complicated extraction or second surgery. Miyashita et al. reported on an origami‐based robot packaged within an ice capsule that is designed to deliver drugs to wounds or remove foreign objects from the stomach.^[^ ^183^ ^]^ The robot unfolds upon melting of the ice capsule and propagates remotely controlled by an external electric field (**Figure** **12**a). A small neodym magnet, attached to the robot, translates the input of the external magnetic field into motion, resulting in a walking speed of 3.7 cm s^−1^. Drug delivery is accomplished upon biodegradation of the origami structure, which starts to degrade after the first 3 h of operation in gastric fluid. Following these results, several groups collaborated to design a deployable hydrogel patch and plug for stomach ulcer therapy.^[^ ^9^ ^]^ In this work, the unfolding of an origami structure is controlled by the rapid swelling of an agarose hydrogel, once the robot is at the ulcer site in the stomach. Similar to the previous concept, the authors utilized a neodym magnet at the center of the robot to achieve the remotely controlled deployment. Introducing adhesive layers to the robot would further increase its functionality by establishing a firm contact of the wound patch to the stomach tissue (Figure 12b).

Instead of a single robot, micrometer‐scale swarm robots have large potential in biomedical applications, including drug delivery and diagnostics, due to versatile operation in various body areas and good control of their locomotion and degradation kinetics. Such microswimmers are usually architectured as helical spirals that rotate upon application of an external magnetic field (for details see^[^ ^184^, ^185^ ^]^) (Figure 12c). Incorporating drugs into the host matrix allows precise deployment to the targeted areas through tuning the degradation and propagation times accordingly. Alternatively, the drug release can be triggered on‐demand by an external light stimulus, as demonstrated by Bozuyuk and co‐workers.^[^ ^186^ ^]^ They fabricated double‐helical microswimmers (6 µm diameter and 20 µm length) from synthetic methacrylated chitosan and modified the side groups to be photocleavable. Irradiation with UV‐light resulted in a release of the chemotherapeutic drug doxorubicin from the microswimmers within 5 min (Figure 12d). Typically, microswimmers are fabricated via two‐photon lithography—a technique that allows 3D‐printing of photo‐crosslinkable polymers on the micrometer scale and below—and modified with magnetic nanoparticles (e.g., F_3_O_4_ nanoparticles) to be receptive to external magnetic fields (Figure 12e). Wang et al. utilized two photon polymerization (2PP) of GelMa to fabricate biodegradable microswimmers in the length range of 25 to 150 µm.^[^ ^187^ ^]^ The microswimmers achieved a maximum forward velocity of 16 µm s^−1^ (and minimal drift velocity) when subject to an 8 mT magnetic field rotating at 16 Hz. The authors note that the soft nature of GelMa contributes favorably to the propagation properties as the forward velocity remains high also at higher frequencies. As GelMa is typically used for cell growth in tissue engineering applications, neuron‐like cells can be attached to microswimmers carrying them to a region of interest, and undergo stimulated cell differentiation.^[^ ^188^ ^]^ For the latter, Dong et al. utilized double‐functional magnetic nanoparticles, which enable propagation when subjected to rotating magnetic fields and electrical stimulation under alternating magnetic fields.

### 6.4 Biohybrid Actuators

For millimeter‐sized robots, which are designed to operate in nonlocal uncontrollable environments, a controlled propagation needs to be solved differently. Biohybrid actuators utilize living cells to enable actuation on a small scale in close analogy to nature and enable biodegradable and biocompatible robotic solutions.

**Figure 13:**
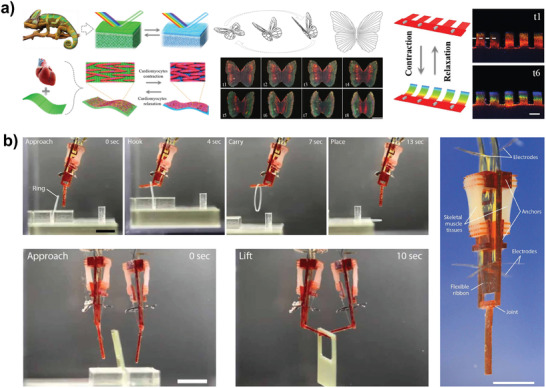
Biohybrid actuators. a) A color changing inverse opal structure is actuated by cardiomyocytes to establish butterfly‐like demonstrations and self‐reporting microfluidic sensors. Scale bar: 1 mm. Reproduced with permission.^[^ ^89^ ^]^ Copyright 2018, The American Association for the Advancement of Science. b) Robotic manipulator based on an antagonistic pair of skeletal muscle tissue can carry small objects on demand. Scale bars: top and bottom left: 1 cm, right: 5 mm. Reproduced with permission.^[^ ^11^ ^]^ Copyright 2018, The American Association for the Advancement of Science.

Fu and co‐workers developed structural color hydrogels from soft inverse opal GelMa hydrogel films, which were operated by engineered cardiomyocyte tissues.^[^ ^89^ ^]^ The explanted cardiomyocytes quickly regained their autonomic beating capability when placed onto the micro structured hydrogel and translated their contraction into a structural color change of the GelMa gel (**Figure** **13**a). The authors utilized this concept for a 2D self‐regulating color pattern and a 3D dynamic butterfly model to demonstrate versatile options for the design of actuators. The first system represents an organ‐on‐a‐chip device where microfluidic channels were used to provide culture medium uniformly to the cardiac muscle cells on the inverse opal hydrogel. The hydrogel was partially fixed to the microfluidic device, which resulted in a bending motion and characteristic color change upon actuation by the cardiac muscle cells. Mixing different concentrations of isoproterenol to culture medium stimulated the beating frequency and caused a blue shift of the color change, making changes in the culture medium immediately visible. In the second application the GelMa hydrogels were prepared in the shape of butterfly wings. The contraction of the cardiac muscle cells bent the wings similar to a real butterfly and resulted in repeated color changes due to a change of the Bragg glancing angle.

While cardiomyocytes have great potential for robotic applications that require a continuous repeated actuation, this cell type is not suitable for applications that need to be switched off and on (see Section 3.4). Here, skeletal muscle tissues are an attractive alternative as their contraction can be precisely controlled, but they suffer from spontaneous shrinkage during cell culture. Morimoto and co‐workers circumvent this problem by culturing an antagonistic pair of skeletal muscle tissues to cancel the intrinsic traction forces.^[^ ^11^ ^]^ The contractions of the individual muscles are electrically stimulated and result in a smooth bidirectional rotation of a joint and extended manipulator. Bending angles of 80° were reliably achieved for the manipulator with an applied electric field of 1.5 V mm^−1^. While there is a tendency that intermediate bending states can be achieved by tuning the stimulation voltage, the variation for a specific voltage input remains large. Nonetheless, the authors achieved to perform pick‐and‐place operations of small objects and demonstrated a gripper‐type device consisting of two individual manipulators (Figure 13b). As the performance of a muscle pair changes similarly over time, the manipulators remained operational for about a week, without notable spontaneous shrinkage. More complex systems are readily achievable as long as antagonistic muscle arrangements are maintained and can lead to versatile soft robots based on biohybrid actuators.

## 7 Conclusion

With the rise of sustainable material approaches for electronic devices, nature‐inspired forms of soft and lightweight robots emerge, which almost exclusively employ renewable, ecofriendly, and biodegradable components. The diverse soft robotic creations benefit manifold from these activities, as a careful use of resources reduces weight and promotes autonomous operation, bioresorbable materials allow in vivo deployment, or biodegradation eliminates waste issues and usage in remote areas without worries. At the forefront of materials research scientists aim to unite performance and sustainability while keeping materials and production costs low, to allow ready implementation in state‐of‐the art prototypes. Highly stretchable yet biodegradable polymers, transient sensors and transistors, and easy to recycle batteries assembled in ecofriendly fabrication lines are examples of major interdisciplinary research goals covering diverse fields. Beyond the development of individual components, the tight integration into fully autonomous robots is difficult to achieve, which requires to rethink current robot designs, actuation principles, and energy supply. This must also include concepts for their repair, disassembly, reuse, and refabrication, which all benefit from designs with reduced complexity, self‐healing, or biodegradable components. Integrating sustainability as a key metric in our future creations will not only become a must but also open a multitude of possible applications across diverse ecological niches. Renewable, low‐cost and easy to access resources finally will allow for low energy production of tech‐products and simpler recycling schemes. However, achieving this will require an extensive amount of research dedicated to closing the gap to high performance solutions or provide entirely new pathways—with robotics as the major driver toward sustainable technology development—that mitigate this challenge.

## Conflict of Interest

The authors declare no conflict of interest.

## References

[1] C. P. Balde , V. Forti , V. Gray , R. Kuehr , P. Stegmann , The Global E‐Waste Monitor ‐ 2017, United Nations University (UNU), International Telecommunication Union (ITU) & International Solid Waste Association (ISWA), Bonn/Geneva/Vienna 2017.

[2] E. Awere , P. A. Obeng , A. Bonoli , P. A. Obeng , Environ. Technol. Rev. 2020, 9, 1.

[3] M. Gao , C. C. Shih , S. Y. Pan , C. C. Chueh , W. C. Chen , J. Mater. Chem. A 2018, 6, 20546.

[4] G. Z. Yang , J. Bellingham , P. E. Dupont , P. Fischer , L. Floridi , R. Full , N. Jacobstein , V. Kumar , M. McNutt , R. Merrifield , B. J. Nelson , B. Scassellati , M. Taddeo , R. Taylor , M. Veloso , Z. L. Wang , R. Wood , Sci. Rob. 2018, 3, eaar7650. 10.1126/scirobotics.aar7650 33141701

[5] C. Majidi , Adv. Mater. Technol. 2019, 4, 1800477.

[6] L. Shui , L. Zhu , Z. Yang , Y. Liu , X. Chen , Soft Matter 2017, 13, 8223. 29083008 10.1039/c7sm01617d

[7] B. Mazzolai , C. Laschi , Sci. Rob. 2020, 5, eaba6893 10.1126/scirobotics.aba6893 33022592

[8] M. Baumgartner , F. Hartmann , M. Drack , D. Preninger , D. Wirthl , R. Gerstmayr , L. Lehner , G. Mao , R. Pruckner , S. Demchyshyn , L. Reiter , M. Strobel , T. Stockinger , D. Schiller , S. Kimeswenger , F. Greibich , G. Buchberger , E. Bradt , S. Hild , S. Bauer , M. Kaltenbrunner , Nat. Mater. 2020, 10.1038/s41563-020-0699-3. 32541932

[9] A. D. P. D'Argentre , S. Perry , Y. Iwata , H. Iwasaki , E. Iwase , A. Fabozzo , I. Will , D. Rus , D. D. Damian , S. Miyashita , in Proc. ‐ IEEE Int. Conf. on Robotics And Automation, IEEE, Piscataway, NJ 2018, pp. 1511–1518.

[10] I. C. Yasa , H. Ceylan , U. Bozuyuk , A.‐M. Wild , M. Sitti , Sci. Rob. 2020, 5, eaaz3867. 10.1126/scirobotics.aaz3867 33022620

[11] Y. Morimoto , H. Onoe , S. Takeuchi , Sci. Rob. 2018, 3, aat440. 10.1126/scirobotics.aat4440 33141706

[12] A. A. La Mattina , S. Mariani , G. Barillaro , Adv. Sci. 2020, 7, 1902872. 10.1002/advs.201902872 PMC7029671 32099766

[13] E. Fan , L. Li , Z. Wang , J. Lin , Y. Huang , Y. Yao , R. Chen , F. Wu , Chem. Rev. 2020, 120, 7020. 31990183 10.1021/acs.chemrev.9b00535

[14] M. Kaltenbrunner , M. S. White , E. D. Głowacki , T. Sekitani , T. Someya , N. S. Sariciftci , S. Bauer , Nat. Commun. 2012, 3, 770. 22473014 10.1038/ncomms1772 PMC3337988

[15] G. Koronis , A. Silva , M. Fontul , Composites, Part B 2013, 44, 120.

[16] C. Wang , K. Xia , H. Wang , X. Liang , Z. Yin , Y. Zhang , Adv. Mater. 2019, 31, 1801072. 10.1002/adma.201801072 30300444

[17] H. Hou , X. Qiu , W. Wei , Y. Zhang , X. Ji , Adv. Energy Mater. 2017, 7, 1602898.

[18] Nat. Energy 2019, 4, 253, 10.1038/s41560-019-0376-4.

[19] Y. J. Tan , H. Godaba , G. Chen , S. T. M. Tan , G. Wan , G. Li , P. M. Lee , Y. Cai , S. Li , R. F. Shepherd , J. S. Ho , B. C. K. Tee , Nat. Mater. 2020, 19, 182. 31844282 10.1038/s41563-019-0548-4

[20] J. M. Allwood , J. M. Cullen , M. A. Carruth , D. R. Cooper , M. McBrien , R. L. Milford , M. C. Moynihan , A. C. Patel , UIT Cambridge Limited, Cambridge, UK 2012, p. 3.

[21] V. R. Feig , H. Tran , Z. Bao , ACS Cent. Sci. 2018, 4, 337. 29632879 10.1021/acscentsci.7b00595 PMC5879474

[22] A. R. Bagheri , C. Laforsch , A. Greiner , S. Agarwal , Global Challenges 2017, 1, 1700048. 31565274 10.1002/gch2.201700048 PMC6607129

[23] B. Rieger , A. Künkel , G. W. Coates , R. Reichardt , E. Dinjus , T. A. Zevaco , Synthetic Biodegradable Polymers, Springer Science & Business Media, New York 2012.

[24] G. M. Whitesides , Angew. Chem., ‐ Int. Ed. 2018, 57, 4258. 10.1002/anie.201800907 29517838

[25] Z. Taylor , K. Miller , J. Biomech. 2004, 37, 1263. 15212932 10.1016/j.jbiomech.2003.11.027

[26] S. Diridollou , V. Vabre , M. Berson , L. Vaillant , D. Black , J. M. Lagarde , J. M. Grégoire , Y. Gall , F. Patat , Int. J. Cosmet. Sci. 2001, 23, 353. 18498486 10.1046/j.0412-5463.2001.00105.x

[27] S. Bohm , F. Mersmann , M. Tettke , M. Kraft , A. Arampatzis , J. Exp. Biol. 2014, 217, 4010. 25267851 10.1242/jeb.112268

[28] T. S. Keller , Z. Mao , D. M. Spengler , J. Orthop. Res. 1990, 8, 592. 2355299 10.1002/jor.1100080416

[29] Y. Kikkawa , S. Tanaka , Y. Norikane , RSC Adv. 2017, 7, 55720.

[30] J. Vaicekauskaite , P. Mazurek , S. Vudayagiri , A. L. Skov , J. Mater. Chem. C 2020, 8, 1273.

[31] R. Moser , G. Kettlgruber , C. M. Siket , M. Drack , I. M. Graz , U. Cakmak , Z. Major , M. Kaltenbrunner , S. Bauer , Adv. Sci. 2016, 3, 1500396. 10.1002/advs.201500396 PMC4985695 27588259

[32] Q. Chen , S. Liang , G. A. Thouas , Prog. Polym. Sci. 2013, 38, 584.

[33] J. D. Fromstein , K. A. Woodhouse , J. Biomater. Sci., Polym. Ed. 2002, 13, 391. 12160300 10.1163/156856202320253929

[34] G. A. Skarja , K. A. Woodhouse , J. Biomater. Sci., Polym. Ed. 2001, 12, 851. 11718481 10.1163/156856201753113060

[35] H. M. Dou , J. H. Ding , H. Chen , Z. Wang , A. F. Zhang , H. Bin Yu , RSC Adv. 2019, 9, 13104. 35520808 10.1039/c9ra01583c PMC9063761

[36] C. J. Bettinger , Macromol. Biosci. 2011, 11, 467. 21229578 10.1002/mabi.201000397

[37] Y. Wang , G. A. Ameer , B. J. Sheppard , R. Langer , Nat. Biotechnol. 2002, 20, 602. 12042865 10.1038/nbt0602-602

[38] S. Walker , J. Rueben , T. Van Volkenburg , S. Hemleben , C. Grimm , J. Simonsen , Y. Mengüç , Int. J. Intell. Rob. Appl. 2017, 1, 124.

[39] J. Rueben , S. Walker , S. Huhn , J. Simonsen , Y. Mengüç , MRS Adv. 2018, 3, 1551.

[40] M. J. Kim , M. Y. Hwang , J. Kim , D. J. Chung , Biomed Res. Int. 2014, 2014, 956952. 24955369 10.1155/2014/956952 PMC4052888

[41] A. R. Webb , J. Yang , G. A. Ameer , Expert Opin. Biol. Ther. 2004, 4, 801. 15174963 10.1517/14712598.4.6.801

[42] J. Yang , A. R. Webb , G. A. Ameer , Adv. Mater. 2004, 16, 511.

[43] R. T. Tran , P. Thevenot , D. Gyawali , J. C. Chiao , L. Tang , J. Yang , Soft Matter 2010, 6, 2449. 22162975 10.1039/C001605E PMC3233194

[44] C. M. Boutry , Y. Kaizawa , B. C. Schroeder , A. Chortos , A. Legrand , Z. Wang , J. Chang , P. Fox , Z. Bao , Nat. Electron. 2018, 1, 314.

[45] K. Y. Lee , D. J. Mooney , Prog. Polym. Sci. 2012, 37, 106. 22125349 10.1016/j.progpolymsci.2011.06.003 PMC3223967

[46] O. Jeon , K. H. Bouhadir , J. M. Mansour , E. Alsberg , Biomaterials 2009, 30, 2724. 19201462 10.1016/j.biomaterials.2009.01.034

[47] E. Kim , M. H. Kim , J. H. Song , C. Kang , W. H. Park , Int. J. Biol. Macromol. 2020, 154, 989. 32194119 10.1016/j.ijbiomac.2020.03.134

[48] J. Y. Sun , X. Zhao , W. R. K. Illeperuma , O. Chaudhuri , K. H. Oh , D. J. Mooney , J. J. Vlassak , Z. Suo , Nature 2012, 489, 133. 22955625 10.1038/nature11409 PMC3642868

[49] C. Zhu , R. Yang , X. Hua , H. Chen , J. Xu , R. Wu , L. Cen , J. Biomater. Sci., Polym. Ed. 2018, 29, 543. 29316854 10.1080/09205063.2018.1426425

[50] T. N. Tran , A. Athanassiou , A. Basit , I. S. Bayer , Food Chem. 2017, 216, 324. 27596427 10.1016/j.foodchem.2016.08.055

[51] L. Averous , L. Moro , P. Dole , C. Fringant , Polymer 2000, 41, 4157.

[52] L. Ceseracciu , J. A. Heredia‐Guerrero , S. Dante , A. Athanassiou , I. S. Bayer , ACS Appl. Mater. Interfaces 2015, 7, 3742. 25622232 10.1021/am508515z

[53] Allevi Bioprinting , https://www.allevi3d.com/ (accessed: May 2020).

[54] Cellink Life Sciences , https://www.cellink.com (accessed: May 2020).

[55] A. I. Van Den Bulcke , B. Bogdanov , N. De Rooze , E. H. Schacht , M. Cornelissen , H. Berghmans , Biomacromolecules 2000, 1, 31. 11709840 10.1021/bm990017d

[56] Y. Wang , M. Ma , J. Wang , W. Zhang , W. Lu , Y. Gao , B. Zhang , Y. Guo , Materials 2018, 11, 6.

[57] T. Wu , Z. Xu , Y. Zhang , H. Wang , C. Cui , B. Chang , X. Feng , W. Liu , Macromol. Mater. Eng. 2018, 303, 1800290.

[58] J. Shintake , H. Sonar , E. Piskarev , J. Paik , D. Floreano , IEEE Int. Conf. Intell. Rob. Syst. 2017, 6221, 10.1109/IROS.2017.8206525.

[59] Q. He , Y. Huang , S. Wang , Adv. Funct. Mater. 2018, 28, 1705069.

[60] Z. Qin , D. Dong , M. Yao , Q. Yu , X. Sun , Q. Guo , H. Zhang , F. Yao , J. Li , ACS Appl. Mater. Interfaces 2019, 11, 21184. 31117467 10.1021/acsami.9b05652

[61] R. Scaffaro , A. Maio , F. Sutera , E. ortunato Gulino , M. Morreale , Polymers 2019, 11, 651. 30970659 10.3390/polym11040651 PMC6523205

[62] N. R. Nair , V. C. Sekhar , K. M. Nampoothiri , A. Pandey , Biodegradation of Biopolymers, Elsevier B.V., Amsterdam 2016.

[63] J. Seppälä , T. Karjalainen , M. Hiljanen‐Vainio , J. Appl. Polym. Sci 1996, 59, 1281.

[64] D. P. Martin , S. F. Williams , Biochem. Eng. J. 2003, 16, 97.

[65] M. A. Woodruff , D. W. Hutmacher , Prog. Polym. Sci. 2010, 35, 1217.

[66] A. S. Al Hosni , J. K. Pittman , G. D. Robson , Waste Manage. 2019, 97, 105. 10.1016/j.wasman.2019.07.042 31447017

[67] M. Vert , J. Mater. Sci.: Mater. Med. 2009, 20, 437. 18815731 10.1007/s10856-008-3581-4

[68] S. Ansari , T. Fatma , PLoS One 2016, 11, e0158168. 27359097 10.1371/journal.pone.0158168 PMC4928839

[69] C. S. K. Reddy , R. Ghai , Rashmi , V. C. Kalia , Bioresour. Technol. 2003, 87, 137. 12765352 10.1016/s0960-8524(02)00212-2

[70] Q. Zhao , Y. Ding , B. Yang , N. Ning , Q. Fu , Polym. Test. 2013, 32, 299.

[71] L. T. Sin , B. S. Tueen , Polylactic Acid: A Practical Guide for the Processing, Manufacturing, and Applications of PLA, 2nd ed., Elsevier, Amsterdam 2019.

[72] E. Vey , C. Rodger , J. Booth , M. Claybourn , A. F. Miller , A. Saiani , Polym. Degrad. Stab. 2011, 96, 1882.

[73] S. A. Park , H. Jeon , H. Kim , S. H. Shin , S. Choy , D. S. Hwang , J. M. Koo , J. Jegal , S. Y. Hwang , J. Park , D. X. Oh , Nat. Commun. 2019, 10, 1. 31197142 10.1038/s41467-019-10582-6 PMC6565616

[74] P. Cazón , G. Velazquez , J. A. Ramírez , M. Vázquez , Food Hydrocolloids 2017, 68, 136.

[75] W. A. Laftah , Int. J. Eng. Res. Technol. 2017, 6, 1151.

[76] O. Lopez , M. A. Garcia , M. A. Villar , A. Gentili , M. S. Rodriguez , L. Albertengo , LWT ‐ Food Sci. Technol. 2014, 57, 106.

[77] A. P. Martínez‐Camacho , M. O. Cortez‐Rocha , J. M. Ezquerra‐Brauer , A. Z. Graciano‐Verdugo , F. Rodriguez‐Félix , M. M. Castillo‐Ortega , M. S. Yépiz‐Gómez , M. Plascencia‐Jatomea , Carbohydr. Polym. 2010, 82, 305. 10.1016/j.carbpol.2012.08.076 23121962

[78] R. V. Gadhave , A. Das , P. A. Mahanwar , P. T. Gadekar , Open J. Polym. Chem. 2018, 08, 21.

[79] F. Hoeng , A. Denneulin , J. Bras , Nanoscale 2016, 8, 13131. 27346635 10.1039/c6nr03054h

[80] H. Kargarzadeh , M. Mariano , J. Huang , N. Lin , I. Ahmad , A. Dufresne , S. Thomas , Polymer 2017, 132, 368.

[81] L. K. Rivera‐Tarazona , V. D. Bhat , H. Kim , Z. T. Campbell , T. H. Ware , Sci. Adv. 2020, 6, eaax8582. 32010767 10.1126/sciadv.aax8582 PMC6968942

[82] E. Del Dottore , A. Sadeghi , A. Mondini , V. Mattoli , B. Mazzolai , Front. Rob. AI 2018, 5, 16. 10.3389/frobt.2018.00016 PMC7805952 33500903

[83] A. Sadeghi , A. Mondini , B. Mazzolai , Soft Rob. 2017, 4, 211. 10.1089/soro.2016.0080 PMC5649421 29062628

[84] L. Ricotti , B. Trimmer , A. W. Feinberg , R. Raman , K. K. Parker , R. Bashir , M. Sitti , S. Martel , P. Dario , A. Menciassi , Sci. Rob. 2017, 2, eaaq0495. 10.1126/scirobotics.aaq0495 33157905

[85] M. Jones , A. Mautner , S. Luenco , A. Bismarck , S. John , Mater. Des. 2020, 187, 108397.

[86] M. Haneef , L. Ceseracciu , C. Canale , I. S. Bayer , J. A. Heredia‐Guerrero , A. Athanassiou , Sci. Rep. 2017, 7, 41292. 28117421 10.1038/srep41292 PMC5259796

[87] M. R. Islam , G. Tudryn , R. Bucinell , L. Schadler , R. C. Picu , Sci. Rep. 2017, 7, 13070. 29026133 10.1038/s41598-017-13295-2 PMC5638950

[88] M. P. Jones , A. C. Lawrie , T. T. Huynh , P. D. Morrison , A. Mautner , A. Bismarck , S. John , Process Biochem. 2019, 80, 95.

[89] F. Fu , L. Shang , Z. Chen , Y. Yu , Y. Zhao , Sci. Rob. 2018, 3, eaar8580. 10.1126/scirobotics.aar8580 33141750

[90] S. R. Shin , C. Shin , A. Memic , S. Shadmehr , M. Miscuglio , H. Y. Jung , S. M. Jung , H. Bae , A. Khademhosseini , X. Tang , M. R. Dokmeci , Adv. Funct. Mater. 2015, 25, 4486. 27134620 10.1002/adfm.201501379 PMC4849195

[91] S.‐J. Park , M. Gazzola , K. S. Park , S. Park , V. Di Santo , E. L. Blevins , J. U. Lind , P. H. Campbell , S. Dauth , A. K. Capulli , F. S. Pasqualini , S. Ahn , A. Cho , H. Yuan , B. M. Maoz , R. Vijaykumar , J.‐W. Choi , K. Deisseroth , G. V Lauder , L. Mahadevan , K. K. Parker , Science 2016, 353, 158 LP. 27387948 10.1126/science.aaf4292 PMC5526330

[92] J. Kim , J. Park , S. Yang , J. Baek , B. Kim , S. H. Lee , E. S. Yoon , K. Chun , S. Park , Lab Chip 2007, 7, 1504. 17960278 10.1039/b705367c

[93] Y. Akiyama , K. Iwabuchi , Y. Furukawa , K. Morishima , Lab Chip 2009, 9, 140. 19209346 10.1039/b809299k

[94] Y. Choi , J. Koo , J. A. Rogers , MRS Bull. 2020, 45, 103.

[95] L. Yin , H. Cheng , S. Mao , R. Haasch , Y. Liu , X. Xie , S.‐W. Hwang , H. Jain , S.‐K. Kang , Y. Su , R. Li , Y. Huang , J. A. Rogers , Adv. Funct. Mater. 2014, 24, 645.

[96] Y. F. Zheng , X. N. Gu , F. Witte , Mater. Sci. Eng., R 2014, 77, 1.

[97] G. Song , A. Atrens , Adv. Eng. Mater. 2003, 5, 837.

[98] R. Ambat , N. N. Aung , W. Zhou , J. Appl. Electrochem. 2000, 30, 865.

[99] E. Hsu , K. Barmak , A. C. West , A. H. A. Park , Green Chem. 2019, 21, 919.

[100] Z. Ma , D. Kong , L. Pan , Z. Bao , J. Semicond. 2020, 41, 41601.

[101] X. Liu , M. Shi , Y. Luo , L. Zhou , Z. R. Loh , Z. J. Oon , X. Lian , X. Wan , F. B. L. Chong , Y. Tong , Appl. Sci. 2020, 10, 1320.

[102] L. Wang , K. Wang , Z. Lou , K. Jiang , G. Shen , Adv. Funct. Mater. 2018, 28, 1804510.

[103] M. Irimia‐Vladu , P. A. Troshin , M. Reisinger , L. Shmygleva , Y. Kanbur , G. Schwabegger , M. Bodea , R. Schwödiauer , A. Mumyatov , J. W. Fergus , V. F. Razumov , H. Sitter , N. S. Sariciftci , S. Bauer , Adv. Funct. Mater. 2010, 20, 4069.

[104] C. J. Bettinger , Z. Bao , Adv. Mater. 2010, 22, 651. 20217767 10.1002/adma.200902322 PMC2868598

[105] S.‐W. Hwang , H. Tao , D.‐H. Kim , H. Cheng , J.‐K. Song , E. Rill , M. A. Brenckle , B. Panilaitis , S. M. Won , Y.‐S. Kim , Y. M. Song , K. J. Yu , A. Ameen , R. Li , Y. Su , M. Yang , D. L. Kaplan , M. R. Zakin , M. J. Slepian , Y. Huang , F. G. Omenetto , J. A. Rogers , Science 2012, 337, 1640 LP. 23019646 10.1126/science.1226325 PMC3786576

[106] G. D. Cha , D. Kang , J. Lee , D.‐H. Kim , Adv. Healthcare Mater. 2019, 8, 1801660.

[107] P. Delmas , J. Hao , L. Rodat‐Despoix , Nat. Rev. Neurosci. 2011, 12, 139. 21304548 10.1038/nrn2993

[108] C. Dhong , R. Miller , N. B. Root , S. Gupta , L. V Kayser , C. W. Carpenter , K. J. Loh , V. S. Ramachandran , D. J. Lipomi , Sci. Adv. 2019, 5, eaaw8845. 31497646 10.1126/sciadv.aaw8845 PMC6716960

[109] L. Gao , C. Zhu , L. Li , C. Zhang , J. Liu , H. D. Yu , W. Huang , ACS Appl. Mater. Interfaces 2019, 11, 25034. 31268663 10.1021/acsami.9b07465

[110] T. D. Nguyen , E. J. Curry , in 2019 IEEE 16th Int. Conf. on Wearable and Implantable Body Sensors Networks, BSN 2019 ‐ Proc., IEEE, Piscataway, NJ 2019, p. 19.

[111] E. J. Curry , T. T. Le , R. Das , K. Ke , E. M. Santorella , D. Paul , M. T. Chorsi , K. T. M. Tran , J. Baroody , E. R. Borges , B. Ko , A. Golabchi , X. Xin , D. Rowe , L. Yue , J. Feng , M. Daniela Morales‐Acosta , Q. Wu , I. P. Chen , X. Tracy Cui , J. Pachter , T. D. Nguyen , Proc. Natl. Acad. Sci. USA 2020, 117, 214. 31871178 10.1073/pnas.1910343117 PMC6955346

[112] M. A. U. Khalid , M. Ali , A. M. Soomro , S. W. Kim , H. B. Kim , B. G. Lee , K. H. Choi , Sensors Actuators, A 2019, 294, 140.

[113] C. Hou , Z. Xu , W. Qiu , R. Wu , Y. Wang , Q. Xu , X. Y. Liu , W. Guo , Small 2019, 15, 1805084. 10.1002/smll.201805084 30690886

[114] C. Wang , X. Li , E. Gao , M. Jian , K. Xia , Q. Wang , Z. Xu , T. Ren , Y. Zhang , Adv. Mater. 2016, 28, 6640. 27168096 10.1002/adma.201601572

[115] S. R. A. Ruth , L. Beker , H. Tran , V. R. Feig , N. Matsuhisa , Z. Bao , Adv. Funct. Mater. 2019, 30, 1903100.

[116] C. M. Boutry , A. Nguyen , Q. O. Lawal , A. Chortos , S. Rondeau‐Gagné , Z. Bao , Adv. Mater. 2015, 27, 6954. 26418964 10.1002/adma.201502535

[117] C. M. Boutry , M. Negre , M. Jorda , O. Vardoulis , A. Chortos , O. Khatib , Z. Bao , Sci. Rob. 2018, 3, eaau6914. 10.1126/scirobotics.aau6914 33141713

[118] G. A. Salvatore , J. Sülzle , F. Dalla Valle , G. Cantarella , F. Robotti , P. Jokic , S. Knobelspies , A. Daus , L. Büthe , L. Petti , N. Kirchgessner , R. Hopf , M. Magno , G. Tröster , Adv. Funct. Mater. 2017, 27, 1702390.

[119] S. M. Won , J. Koo , K. E. Crawford , A. D. Mickle , Y. Xue , S. Min , L. A. McIlvried , Y. Yan , S. B. Kim , S. M. Lee , B. H. Kim , H. Jang , M. R. MacEwan , Y. Huang , R. W. Gereau IV , J. A. Rogers , Adv. Funct. Mater. 2018, 28, 1801819.

[120] F. Güder , A. Ainla , J. Redston , B. Mosadegh , A. Glavan , T. J. Martin , G. M. Whitesides , Angew. Chem., Int. Ed. 2016, 55, 5727. 10.1002/anie.201511805 27059088

[121] A. Burton , S. N. Obaid , A. Vázquez‐Guardado , M. B. Schmit , T. Stuart , L. Cai , Z. Chen , I. Kandela , C. R. Haney , E. A. Waters , H. Cai , J. A. Rogers , L. Lu , P. Gutruf , Proc. Natl. Acad. Sci. USA 2020, 117, 2835 LP. 31974306 10.1073/pnas.1920073117 PMC7022161

[122] H. Zhu , Z. Xiao , D. Liu , Y. Li , N. J. Weadock , Z. Fang , J. Huang , L. Hu , Energy Environ. Sci. 2013, 6, 2105.

[123] E. F. Gomez , A. J. Steckl , ACS Photonics 2015, 2, 439.

[124] N. Jürgensen , M. Ackermann , T. Marszalek , J. Zimmermann , A. J. Morfa , W. Pisula , U. H. F. Bunz , F. Hinkel , G. Hernandez‐Sosa , ACS Sustainable Chem. Eng. 2017, 5, 5368.

[125] D. Lu , T.‐L. Liu , J.‐K. Chang , D. Peng , Y. Zhang , J. Shin , T. Hang , W. Bai , Q. Yang , J. A. Rogers , Adv. Mater. 2019, 31, 1902739. 10.1002/adma.201902739 31489737

[126] X. Jia , C. Wang , C. Y. Lee , C. Yu , G. G. Wallace , MRS Bull. 2020, 45, 121.

[127] J.‐K. Chang , H. Fang , C. A. Bower , E. Song , X. Yu , J. A. Rogers , Proc. Natl. Acad. Sci. USA 2017, 114, E5522 LP. 28652373 10.1073/pnas.1707849114 PMC5514770

[128] T. Lei , M. Guan , J. Liu , H.‐C. Lin , R. Pfattner , L. Shaw , A. F. McGuire , T.‐C. Huang , L. Shao , K.‐T. Cheng , J. B.‐H. Tok , Z. Bao , Proc. Natl. Acad. Sci. USA 2017, 114, 5107 LP. 28461459 10.1073/pnas.1701478114 PMC5441761

[129] F. Sugiyama , A. T. Kleinschmidt , L. V. Kayser , M. A. Alkhadra , J. M. H. Wan , A. S. C. Chiang , D. Rodriquez , S. E. Root , S. Savagatrup , D. J. Lipomi , Macromolecules 2018, 51, 5944. 30930487 10.1021/acs.macromol.8b00846 PMC6435287

[130] H. Tran , V. R. Feig , K. Liu , H. C. Wu , R. Chen , J. Xu , K. Deisseroth , Z. Bao , ACS Cent. Sci. 2019, 5, 1884. 31807690 10.1021/acscentsci.9b00850 PMC6891860

[131] X. Ji , L. Song , S. Zhong , Y. Jiang , K. G. Lim , C. Wang , R. Zhao , J. Phys. Chem. C 2018, 122, 16909.

[132] S. Liu , S. Dong , X. Wang , L. Shi , H. Xu , S. Huang , J. Luo , Nanotechnology 2020, 31, 255204. 32101798 10.1088/1361-6528/ab7a2c

[133] Z. Zou , C. Zhu , Y. Li , X. Lei , W. Zhang , J. Xiao , Sci. Adv. 2018, 4, eaaq0508. 29487912 10.1126/sciadv.aaq0508 PMC5817920

[134] C. A. Aubin , S. Choudhury , R. Jerch , L. A. Archer , J. H. Pikul , R. F. Shepherd , Nature 2019, 571, 51. 31217583 10.1038/s41586-019-1313-1

[135] T. S. Mathis , N. Kurra , X. Wang , D. Pinto , P. Simon , Y. Gogotsi , Adv. Energy Mater. 2019, 9, 1902007.

[136] J. Xu , H. R. Thomas , R. W. Francis , K. R. Lum , J. Wang , B. Liang , J. Power Sources 2008, 177, 512.

[137] M. K. Tran , M.‐T. F. Rodrigues , K. Kato , G. Babu , P. M. Ajayan , Nat. Energy 2019, 4, 339.

[138] T. Liu , Y. Zhang , C. Chen , Z. Lin , S. Zhang , J. Lu , Nat. Commun. 2019, 10, 1965. 31036805 10.1038/s41467-019-09933-0 PMC6488666

[139] H. Zhu , W. Luo , P. N. Ciesielski , Z. Fang , J. Y. Zhu , G. Henriksson , M. E. Himmel , L. Hu , Chem. Rev. 2016, 116, 9305. 27459699 10.1021/acs.chemrev.6b00225

[140] T. C. Nirmale , B. B. Kale , A. J. Varma , Int. J. Biol. Macromol. 2017, 103, 1032. 28554795 10.1016/j.ijbiomac.2017.05.155

[141] J. L. Espinoza‐Acosta , P. I. Torres‐Chávez , J. L. Olmedo‐Martínez , A. Vega‐Rios , S. Flores‐Gallardo , E. A. Zaragoza‐Contreras , J. Energy Chem. 2018, 27, 1422.

[142] G. Milczarek , O. Inganäs , Science 2012, 335, 1468 LP. 22442478 10.1126/science.1215159

[143] W. E. Tenhaeff , O. Rios , K. More , M. A. McGuire , Adv. Funct. Mater. 2014, 24, 86.

[144] N. Delaporte , G. Lajoie , S. Collin‐Martin , K. Zaghib , Sci. Rep. 2020, 10, 3812. 32123203 10.1038/s41598-020-60633-y PMC7052225

[145] L. Yin , X. Huang , H. Xu , Y. Zhang , J. Lam , J. Cheng , J. A. Rogers , Adv. Mater. 2014, 26, 3879. 24652717 10.1002/adma.201306304

[146] X. Huang , D. Wang , Z. Yuan , W. Xie , Y. Wu , R. Li , Y. Zhao , D. Luo , L. Cen , B. Chen , H. Wu , H. Xu , X. Sheng , M. Zhang , L. Zhao , L. Yin , Small 2018, 14, 1800994. 10.1002/smll.201800994 29806124

[147] K. Chayambuka , G. Mulder , D. L. Danilov , P. H. L. Notten , Adv. Energy Mater. 2018, 8, 1800079.

[148] T. Sun , Z. Li , H. Wang , D. Bao , F. Meng , X. Zhang , Angew. Chem., Int. Ed. 2016, 55, 10662. 10.1002/anie.201604519 27485314

[149] Y. J. Kim , W. Wu , S.‐E. Chun , J. F. Whitacre , C. J. Bettinger , Proc. Natl. Acad. Sci. USA 2013, 110, 20912 LP. 24324163 10.1073/pnas.1314345110 PMC3876213

[150] G. Wang , Z. Dai , Y. Guan , P. Dong , L. Wu , Adv. Mech. Eng. 2014, 6, 270537.

[151] T. Selvaraj , V. Perumal , S. F. Khor , L. S. Anthony , S. C. B. Gopinath , N. Muti Mohamed , Mater. Res. Bull. 2020, 126, 110839.

[152] J. Wang , X. Zhang , Z. Li , Y. Ma , L. Ma , J. Power Sources 2020, 451, 227794.

[153] G. Ma , Q. Yang , K. Sun , H. Peng , F. Ran , X. Zhao , Z. Lei , Bioresour. Technol. 2015, 197, 137. 26320018 10.1016/j.biortech.2015.07.100

[154] S.‐Y. Lu , M. Jin , Y. Zhang , Y.‐B. Niu , J.‐C. Gao , C. M. Li , Adv. Energy Mater. 2018, 8, 1702545.

[155] C. Chen , Y. Zhang , Y. Li , J. Dai , J. Song , Y. Yao , Y. Gong , I. Kierzewski , J. Xie , L. Hu , Energy Environ. Sci. 2017, 10, 538.

[156] X. Chen , N. S. Villa , Y. Zhuang , L. Chen , T. Wang , Z. Li , T. Kong , Adv. Energy Mater. 2020, 10, 1902769.

[157] X. L. Wu , T. Wen , H. L. Guo , S. Yang , X. Wang , A. W. Xu , ACS Nano 2013, 7, 3589. 23548083 10.1021/nn400566d

[158] F. Meder , G. A. Naselli , A. Sadeghi , B. Mazzolai , Adv. Mater. 2019, 31, 1905671. 10.1002/adma.201905671 31682053

[159] L. Yang , L. Chang , Y. Hu , M. Huang , Q. Ji , P. Lu , J. Liu , W. Chen , Y. Wu , Adv. Funct. Mater. 2020, 30, 1908842.

[160] M. Kaltenbrunner , G. Adam , E. D. Głowacki , M. Drack , R. Schwödiauer , L. Leonat , D. H. Apaydin , H. Groiss , M. C. Scharber , M. S. White , N. S. Sariciftci , S. Bauer , Nat. Mater. 2015, 14, 1032. 26301766 10.1038/nmat4388

[161] R. Liu , M. Takakuwa , A. Li , D. Inoue , D. Hashizume , K. Yu , S. Umezu , K. Fukuda , T. Someya , Adv. Energy Mater. 2020, 10, 2070090.

[162] D. Lasrado , S. Ahankari , K. Kar , J. Appl. Polym. Sci. 2020, 137, 48959.

[163] F. Brunetti , A. Operamolla , S. Castro‐Hermosa , G. Lucarelli , V. Manca , G. M. Farinola , T. M. Brown , Adv. Funct. Mater. 2019, 29, 1806798.

[164] Z. Fang , H. Zhu , Y. Yuan , D. Ha , S. Zhu , C. Preston , Q. Chen , Y. Li , X. Han , S. Lee , G. Chen , T. Li , J. Munday , J. Huang , L. Hu , Nano Lett. 2014, 14, 765. 24372201 10.1021/nl404101p

[165] A. Hübler , B. Trnovec , T. Zillger , M. Ali , N. Wetzold , M. Mingebach , A. Wagenpfahl , C. Deibel , V. Dyakonov , Adv. Energy Mater. 2011, 1, 1018.

[166] L. Leonat , M. S. White , E. D. Głowacki , M. C. Scharber , T. Zillger , J. Rühling , A. Hübler , N. S. Sariciftci , J. Phys. Chem. C 2014, 118, 16813.

[167] M. Rawat , E. Jayaraman , S. Balasubramanian , S. S. K. Iyer , Adv. Mater. Technol. 2019, 4, 1900184.

[168] H. Li , X. Li , W. Wang , J. Huang , J. Li , S. Huang , B. Fan , J. Fang , W. Song , Sol. Energy 2019, 188, 158.

[169] D. J. Burke , D. J. Lipomi , Energy Environ. Sci. 2013, 6, 2053.

[170] S. Phan , C. K. Luscombe , Trends Chem. 2019, 1, 670.

[171] L. Hong , H. Yao , Z. Wu , Y. Cui , T. Zhang , Y. Xu , R. Yu , Q. Liao , B. Gao , K. Xian , H. Y. Woo , Z. Ge , J. Hou , Adv. Mater. 2019, 31, 1903441. 10.1002/adma.201903441 31392768

[172] C. Santoro , C. Arbizzani , B. Erable , I. Ieropoulos , J. Power Sources 2017, 356, 225. 28717261 10.1016/j.jpowsour.2017.03.109 PMC5465942

[173] Z. Zhu , T. Kin Tam , F. Sun , C. You , Y. H. Percival Zhang , Nat. Commun. 2014, 5, 3026. 24445859 10.1038/ncomms4026

[174] C. Melhuish , I. Ieropoulos , J. Greenman , I. Horsfield , Auton. Rob. 2006, 21, 187.

[175] M. J. McAnulty , V. G. Poosarla , K.‐Y. Kim , R. Jasso‐Chávez , B. E. Logan , T. K. Wood , Nat. Commun. 2017, 8, 15419. 28513579 10.1038/ncomms15419 PMC5442358

[176] Z. J. Ren , Nat. Energy 2017, 2, 17093.

[177] B. E. Logan , R. Rossi , A. Ragab , P. E. Saikaly , Nat. Rev. Microbiol. 2019, 17, 307. 30846876 10.1038/s41579-019-0173-x

[178] H. Philamore , I. Ieropoulos , A. Stinchcombe , J. Rossiter , Soft Rob. 2016, 3, 186.

[179] S. D. Cezan , H. T. Baytekin , B. Baytekin , Soft Rob. 2020, 7, 444. 10.1089/soro.2019.0036 31990639

[180] B. Shin , J. Ha , M. Lee , K. Park , G. H. Park , T. H. Choi , K. J. Cho , H. Y. Kim , Sci. Rob. 2018, 3, eaar2629. 10.1126/scirobotics.aar2629 33141700

[181] Q. Wu , V. Pradeep , X. Liu , in RoboSoft 2018 IEEE Int. Conf. on Soft Robotics, IEEE, Piscataway, NJ 2018, p. 315, 10.1109/ROBOSOFT.2018.8404938.

[182] A. N. Sardesai , X. M. Segel , M. N. Baumholtz , Y. Chen , R. Sun , B. W. Schork , R. Buonocore , K. O. Wagner , H. M. Golecki , MRS Adv. 2018, 3, 3003.

[183] S. Miyashita , S. Guitron , K. Yoshida , S. Li , D. D. Damian , D. Rus , in 2016 Proc. ‐ IEEE Int. Conf. on Robotics and Automation, IEEE, Piscataway, NJ 2016, p. 909, 10.1109/ICRA.2016.7487222.

[184] M. Sitti , D. S. Wiersma , Adv. Mater. 2020, 32, 1906766. 10.1002/adma.201906766 32053227

[185] H. Ceylan , I. C. Yasa , O. Yasa , A. F. Tabak , J. Giltinan , M. Sitti , ACS Nano 2019, 13, 3353. 30742410 10.1021/acsnano.8b09233 PMC6728090

[186] U. Bozuyuk , O. Yasa , I. C. Yasa , H. Ceylan , S. Kizilel , M. Sitti , ACS Nano 2018, 12, 9617. 30203963 10.1021/acsnano.8b05997

[187] X. Wang , X. H. Qin , C. Hu , A. Terzopoulou , X. Z. Chen , T. Y. Huang , K. Maniura‐Weber , S. Pané , B. J. Nelson , Adv. Funct. Mater. 2018, 28, 1.

[188] M. Dong , X. Wang , X.‐Z. Chen , F. Mushtaq , S. Deng , C. Zhu , H. Torlakcik , A. Terzopoulou , X.‐H. Qin , X. Xiao , J. Puigmartí‐Luis , H. Choi , A. P. Pêgo , Q.‐D. Shen , B. J. Nelson , S. Pané , Adv. Funct. Mater. 2020, 30, 1910323.

